# Clinical utility of targeted next-generation sequencing for rapid detection, species differentiation, drug resistance profiling, and prognostic evaluation in *Mycobacterium tuberculosis*: a multi-center retrospective cohort study

**DOI:** 10.3389/fcimb.2026.1818202

**Published:** 2026-08-18

**Authors:** Li Shi, Mingde Ni, Fan Yang, Wei Shi, Xiuwu Liu, Chen Ni, Fang He, Yunhui Huang, Xiaoyun Yang, Yi Pei

**Affiliations:** 1 Department of Tuberculosis, The Affiliated Changsha Central Hospital, Hengyang Medical School, University of South China, Changsha, Hunan, China; 2 Linyi People’s Hospital, Linyi, Shandong, China; 3 Dinfectome Inc., Nanjing, Jiangsu, China; 4 School of Information Engineering, Changsha Medical University, Changsha, Hunan, China; 5 The Second Clinical Medical College of Guangxi Medical University, Nanning, Guangxi, China

**Keywords:** clinical outcomes, drug resistance, nontuberculous mycobacteria, rapid diagnosis, targeted next-generation sequencing, treatment adjustment, tuberculosis

## Abstract

**Background:**

Tuberculosis (TB) remains a global health threat, which is complicated by challenges in distinguishing TB from nontuberculous mycobacteria (NTM) infections and drug resistance. Conventional diagnostics (including culture, Xpert MTB/RIF, AFB smear, T-SPOT.TB, etc.) are limited by inadequate differentiation of TB and NTM, incomplete resistance profiling, and prolonged turnaround times.

**Methods:**

This retrospective multicenter study enrolled 130 patients with suspected mycobacterial infection between August 2023 and June 2024 at two tertiary hospitals in China. Patients were classified as TB (n=91), NTM (n=18), Other (n=17) and Mixed infection (n=4) based on a composite reference standard (final clinical diagnosis) established independently of targeted next-generation sequencing (tNGS) results. Performance of tNGS versus conventional methods was evaluated across different sample types. Diagnostic performance, drug-resistance detection, therapeutic impact and clinical outcomes were analyzed.

**Results:**

The overall agreement of tNGS detection with clinical diagnoses of pathogens was 80.9% in sputum and 71.3% in bronchoalveolar lavage fluid (BALF), exceeding conventional methods (which remained below 55%). Critically, tNGS effectively distinguished TB from NTM, with diagnostic sensitivity of 84.0% (95% CI, 75.0%–90.8%) for TB and 90.5% (95% CI, 69.6%–98.8%) for NTM, and specificity of 100.0% (95% CI, 90.3%–100.0%) for TB and 99.1% (95% CI, 95.0%–100.0%) for NTM. tNGS simultaneously detected resistance-associated variants relevant to 11 antimycobacterial drugs within approximately 24 hours. Treatment regimens for 52 patients (41.3%) were adjusted following tNGS reporting. Of these, 82.7% showed subsequent clinical improvement, however, these outcome data remain descriptive and do not establish causality.

**Conclusions:**

tNGS enables rapid, accurate, and comprehensive diagnosis of mycobacterial infections, reliably differentiates TB from NTM, and provides drug-resistance profiles to inform therapy, with the potential to assist in optimizing clinical management of TB.

## 1 Introduction

Tuberculosis (TB), caused by *Mycobacterium tuberculosis* (MTB), remains one of the leading infectious causes of morbidity and mortality worldwide, and is highly prevalent in China (Deng et al., 2024; Dheda et al., 2024; Chen H. et al., 2025; Xu and Zhao, 2025). Accurate and timely diagnosis is critical for reducing mortality and curbing transmission, yet up to 40% of TB cases are either undiagnosed or detected late, leading to treatment delays and ongoing community transmission (Teo et al., 2019). It is worth noting that nontuberculous mycobacteria (NTM) pulmonary disease often presents with symptoms and imaging findings indistinguishable from TB, posing difficulties for guiding appropriate therapy (Liang et al., 2024), and misdiagnosis of NTM as TB exposes patients to unnecessary anti-TB regimens and diverts resources, whereas failure to recognize TB in favor of NTM perpetuates transmission and worsens outcomes (Feng et al., 2025). Additionally, the challenge is further amplified by drug resistance (Farhat et al., 2024), particularly extensively drug-resistant (XDR-TB), multidrug-resistant (MDR-TB) and rifampicin-resistant (RR-TB) strains, for which in parts of China, the prevalence of MDR-TB has been reported as high as 25% among specific patient groups, far exceeding national averages (Akalu et al., 2024). Inadequate detection of resistance leads to a substantial clinical, epidemiological, and socioeconomic burden of TB, highlighting the urgent need for more accurate and timely diagnostic methods to avoid inappropriate therapy, higher relapse and death rates, and further propagation of resistant strains.

Currently, several diagnostic tools are employed in clinical practice to detect TB and guide patient management, among which mycobacterial culture is regarded as the gold standard for TB diagnosis, offering high specificity and the ability to identify species as well as perform phenotypic drug susceptibility testing (pDST) (Tran et al., 2025). Nucleic acid amplification tests such as Xpert MTB/RIF (Xpert) provide rapid detection of MTB and rifampicin resistance (Rahman et al., 2025). Acid-fast bacilli (AFB) smear microscopy is widely used for its simplicity and speed (Eigner et al., 2025), while immunological assays including T-SPOT.TB (T-SPOT) and tuberculin skin test purified protein derivative (PPD) can aid diagnosis by detecting host immune responses to TB antigens (Yao et al., 2022; Saikaew et al., 2025), supporting identification of infection, especially in latent or paucibacillary cases (Qi et al., 2024).

However, research has demonstrated that existing clinical diagnostic technologies still have significant limitations in practical application. For instance, as the gold standard for TB diagnosis and resistance detection, culture usually requires several weeks to yield results (Naidoo et al., 2024). For AFB, sensitivity is low, particularly in paucibacillary or extrapulmonary TB cases (Wright et al., 1998). Similarly, interferon-gamma release assays (IGRAs) like T-SPOT and PPD are designed to detect immune responses to TB but are unable to distinguish between latent infection and active TB (Pai and Behr, 2016). Although Xpert has been demonstrated to show high sensitivity (over 70%) for TB, its resistance detection spectrum is narrow, typically only reliably detecting rifampicin (RIF) resistance, and it cannot achieve detection of TB co-infection with NTM (Huh et al., 2019). Therefore, limitations of current clinical detection methods underscore the critical need for diagnostic technologies that can rapidly detect TB, accurately distinguish it from NTM, and provide timely information on multidrug resistance.

In recent years, molecular diagnostic approaches have rapidly diversified. Whole-genome sequencing (WGS) provides comprehensive genomic information but remains costly, labor-intensive, and requires cultured isolates, limiting its routine clinical utility (Meehan et al., 2019). Metagenomic next-generation sequencing (mNGS) offers untargeted pathogen detection but is susceptible to host DNA background, shows variable sensitivity for mycobacteria, and typically does not provide reliable drug resistance profiling (Chen et al., 2025b). Unlike WGS, which interrogates the entire bacterial genome, and mNGS, which performs unbiased sequencing of all nucleic acids within a specimen, targeted next-generation sequencing (tNGS) selectively enriches predefined genomic regions using multiplex PCR prior to sequencing. tNGS has been evaluated as a powerful tool in TB diagnostics with high sensitivity and specificity (Gou et al., 2025), offering rapid and accurate detection of TB, along with comprehensive profiling of drug resistance mutations (Chen et al., 2025a; Wang M. et al., 2025). For example, a recent study of bronchoalveolar lavage fluid (BALF) from 181 patients with confirmed TB showed that tNGS could achieve 94.9% sensitivity in detecting TB agents, demonstrating its reliability in clinical application (Wang M. et al., 2025). Meanwhile, tNGS has been found to facilitate NTM species identification and detection of associated resistance genes, enabling more precise diagnosis and tailored treatment strategies (Zhang et al., 2024). In addition, tNGS showed high concordance with WGS, with sensitivity and specificity exceeding 98% for first-line and second-line anti-TB drugs including RIF, isoniazid (INH), and fluoroquinolones (Schwab et al., 2024; Ou et al., 2025).

Although previous studies have compared tNGS with traditional diagnostic methods and evaluated its performance in detecting MTB or predicting drug resistance, there is still a lack of a comprehensive real-world evaluation of tNGS, including comparisons with multiple traditional methods, differentiation between TB and NTM, consistency with phenotypic drug sensitivity tests, as well as assessment of subsequent treatment adjustments and clinical outcomes. To fill this evidence gap, we conducted a multicenter retrospective study aimed at evaluating the clinical application value of tNGS in the diagnostic and therapeutic pathway of mycobacterial infections. Specifically, we compared its diagnostic performance with traditional methods in differentiating TB from NTM, assessed its accuracy in detecting drug resistance, and explored its impact on treatment decisions and clinical outcomes.

## 2 Materials and methods

### 2.1 Study design and participants

#### 2.1.1 Rules of inclusion and exclusion

This retrospective multicenter investigation was conducted at Changsha Central Hospital and Linyi People’s Hospital. From August 2023 to June 2024, patients with suspected MTB pulmonary infection who were continuously admitted to the hospital were all screened according to the unified inclusion and exclusion criteria of the two participating centers. Suspected MTB infection was defined as the presence of clinical manifestations and/or imaging findings suggesting TB or NTM diseases and required microbiological testing for MTB in routine clinical diagnosis, including tNGS and conventional diagnostic reagents. The final diagnosis classification was determined based on the pre-defined composite reference criteria described below. No restrictions were imposed regarding age or gender. The study protocol received approval from the Ethics Committee of Changsha Central Hospital (Approval Number: 2023137) and was executed in compliance with the Declaration of Helsinki. Since the analysis utilized anonymized clinical data, the requirement for informed consent was waived.

**Figure 1:**
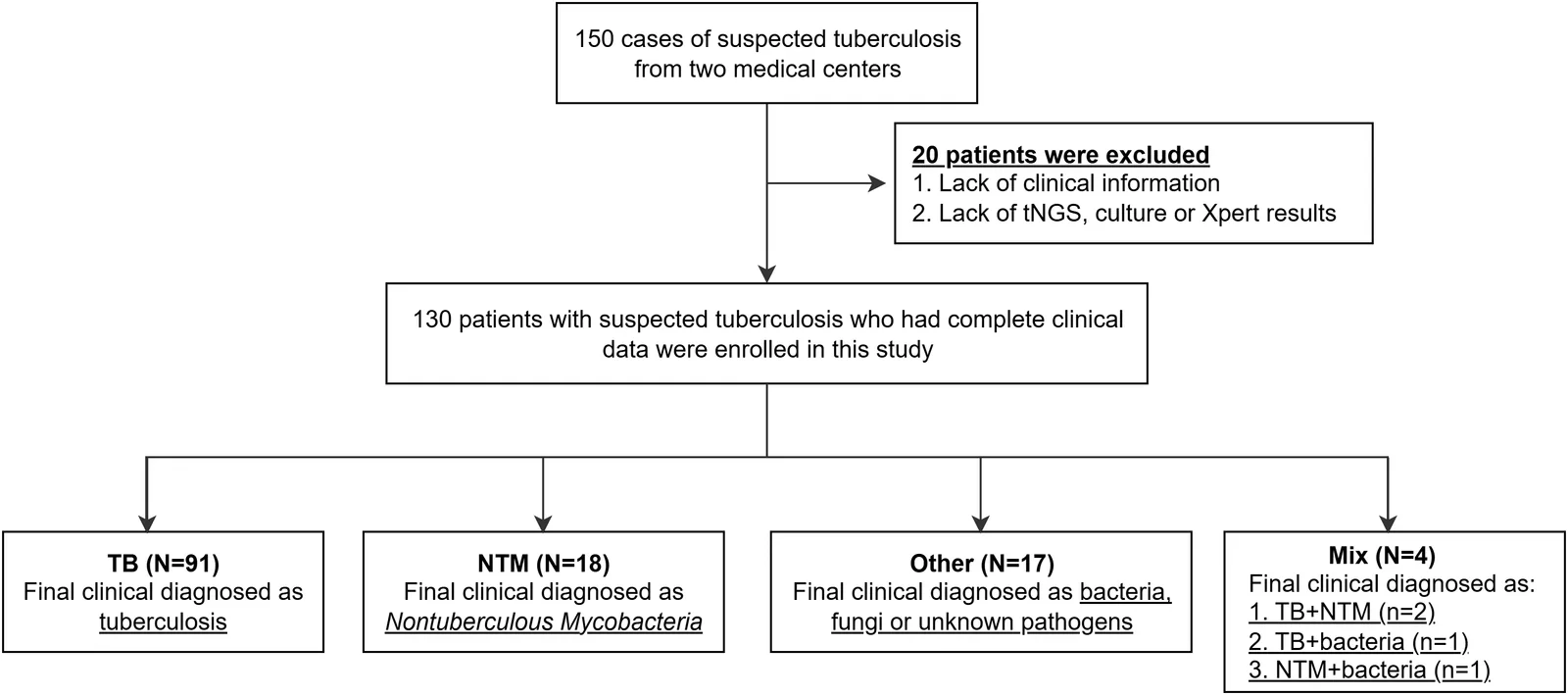
Patient enrollment work flow chart.

A cohort of 150 patients was initially enrolled. Samples lacking complete clinical documentation, tNGS results, culture results, or Xpert MTB/RIF assay results were subsequently excluded. The specific reasons for exclusion were: loss to follow-up with incomplete outcome data (n=12), tNGS library preparation failure (n=5), and missing conventional assay results due to insufficient specimen volume (n=3). Comparison of baseline demographic and clinical characteristics between included (n=130) and excluded (n=20) patients showed no statistically significant differences (**Supplementary Table 1**). Ultimately, 130 samples were included in the final analysis and stratified into the following groups based on the definitive clinical diagnosis (**Figure 1**):

1. TB Group (n = 91): Definitive clinical diagnosis of tuberculosis;
2. NTM Group (n = 18): Definitive clinical diagnosis of nontuberculous mycobacterial disease;
3. Other Group (n = 17): Definitive clinical diagnosis of bacterial infection, fungal infection, or infection with unidentified pathogens;
4. Mixed Infection Group (n = 4): Definitive clinical diagnosis comprised:
5. TB and NTM co-infection (n = 2),
6. TB and bacterial co-infection (n = 1),
7. NTM and bacterial co-infection (n = 1).

#### 2.1.2 Composite reference standard (final clinical diagnosis)

The final clinical diagnosis for each patient was established by two independent senior clinicians (Associate Chief Physician or above) specializing in mycobacterial diseases, who were blinded to all tNGS results. Disagreements were resolved through discussion with a third senior clinician. The diagnostic classification followed predefined criteria, standardized across both study sites:

- TB group: Positive culture for MTB, or positive Xpert MTB/RIF plus consistent clinical and radiological findings plus response to anti-TB therapy, or histopathological evidence of caseating granulomas.
- NTM group: Positive culture for NTM, or molecular species identification plus consistent clinical and radiological features.
- Mixed infection group: Microbiological evidence fulfilling criteria for both TB and NTM, or for one mycobacterial species plus another bacterial/fungal pathogen, with clinical evidence of active co-infection.
- Other group: Alternative microbiologically confirmed diagnosis (bacterial or fungal infection), or clinical diagnosis of non-mycobacterial disease with documented response to non-anti-TB therapy, or no definitive evidence of mycobacterial infection after comprehensive workup.

### 2.2 Clinical and sample collection

Clinical data of enrolled patients were retrospectively collected from hospital records, encompassing demographic characteristics (gender and age), tuberculosis (TB) history, clinical manifestations, imaging features, laboratory indices, specimen types, microbiological results, therapeutic regimens, and clinical outcomes.

All specimens underwent tNGS, culture, and Xpert MTB/RIF testing as part of routine clinical practice, with corresponding results retrospectively extracted from medical records. Respiratory specimens primarily comprised sputum and bronchoalveolar lavage fluid (BALF). Non-respiratory specimens included stool, purulent fluid, urine, pleural effusion, cerebrospinal fluid, pericardial effusion, among others. The detailed breakdown of specimen types is provided in **Supplementary Table 2**. Additionally, results from other routine clinical investigations—including acid-fast bacilli (AFB) smear microscopy, T-SPOT.TB assay, and purified protein derivative (PPD) skin testing—were reviewed as supplementary diagnostic information when available in the medical records.

### 2.3 Strain identification and drug resistance profiling

TB-positive and NTM-positive specimens, categorized by clinical diagnosis, were cultured for pDST and compared with tNGS results. pDST was performed using the MGIT 960 automated liquid culture system (Becton Dickinson, Sparks, MD, USA) according to the manufacturer’s instructions. Critical concentrations for first-line drugs were interpreted following WHO-recommended guidelines: RIF 1.0 μg/mL, INH 0.1 μg/mL (low) and 0.4 μg/mL (high), EMB 5.0 μg/mL, and SM 1.0 μg/mL. For second-line drugs, critical concentrations were: AMK 1.0 μg/mL, KAN 2.5 μg/mL, MFX 0.25 μg/mL (low) and 2.0 μg/mL (high), LFX 1.5 μg/mL, PTH 2.5 μg/mL, PAS 2.0 μg/mL (tested at the Hunan Provincial Tuberculosis Reference Laboratory). Not all isolates underwent complete pDST for all drugs. The number of isolates tested per drug is indicated in **Supplementary Table 3**. The tNGS test panel comprised first- and second-line drugs recommended by the WHO, including rifampicin (RIF), isoniazid (INH), ethambutol (EMB), aminoglycosides, fluoroquinolones, and key companion drugs. Turnaround time was recorded for both methodologies, encompassing the interval from culture initiation to pDST reporting and the complete tNGS workflow from specimen collection to result release.

Furthermore, genotypic DST (gDST) incorporated Xpert MTB/RIF (which detects selected mutations in the *rpoB* gene conferring RIF resistance) and tNGS. Resistance-associated variants were interpreted using a curated in-house mycobacterial resistance database developed for the tNGS platform. The module was constructed using the second edition of the WHO *Catalogue of Mutations in Mycobacterium tuberculosis Complex and Their Association with Drug Resistance* (WHO, 2023), ReSeqTB (accessed in 2024), and relevant peer-reviewed literature available through 2024, and was periodically updated to incorporate newly validated resistance-associated variants. Discordant annotations among the WHO Catalogue, ReSeqTB, and peer-reviewed literature were reviewed collectively against the available phenotypic, functional, clinical, and consensus evidence. Variants with insufficient or conflicting evidence were classified as variants of uncertain significance and did not trigger a resistant call. At the sample level, resistance was reported when a validated resistance-associated variant was detected and the predefined sequencing quality-control requirements were met. The resistance module covered 23 antimicrobial agents across 15 pharmacological classes, involving 19 unique resistance-associated genes or loci. Category-level coverage, including the number of agents, including genes/loci, and applicable organisms, is summarized in **Supplementary Table 4**. For RIF resistance, the entire *rpoB* gene was analyzed, including the rifampicin resistance determinant region (RRDR; codons 507–533) and the non-RRDR region, to enhance the detection ability of rare drug resistance mutations.

### 2.4 tNGS workflow and quality control

#### 2.4.1 Nucleic acid extraction

Clinical specimens were subjected to nucleic acid extraction using standardized protocols for both respiratory and non-respiratory samples. Respiratory specimens (sputum, BALF) were first liquefied and decontaminated using the NALC-NaOH method. Non-respiratory specimens from normally sterile sites underwent direct extraction without decontamination. DNA was extracted using the TIANamp Magnetic DNA Kit (Tiangen, Beijing, China) according to the manufacturer’s protocols and automatically extracted by magnetic bead method using the automatic nucleic acid extractor Purifier 16 (GENFINE, Beijing, China). The quantity was assessed using Qubit 4.0 Fluorometers (Thermo Fisher Scientific, Waltham, MA, USA) and Pico488 dsDNA quantification reagent (Lumiprobe, Hunt Valley, MD, USA), ensuring sufficient concentration for library preparation. Extraction-negative controls (nuclease-free water) and positive controls (*M. tuberculosis* H37Rv genomic DNA) were included in each extraction run.

#### 2.4.2 Multiplex PCR amplification

Multiplex PCR amplification was performed to enrich genomic regions used for mycobacterial identification and clinically relevant resistance-associated loci covered by the assay. The pharmacological class-level coverage and corresponding resistance-associated genes/loci are summarized in **Supplementary Table 4**. Amplification was performed using Hieff^®^ Robust PCR Master Mix (Yeasen Biotech, Shanghai, China) together with a proprietary multiplex primer panel developed by Dinfectome Inc. for targeted enrichment of mycobacterial genomic regions. Primer design was based on conserved target regions and clinically relevant resistance-associated loci covered by the assay. PCR was performed on a Biorad C1000 Touch PCR instrument (Bio-Rad, USA) following the manufacturer’s protocol. Cycling conditions were initial denaturation at 95 °C for 5 min; 30 cycles of 95 °C for 30 s, 60 °C for 30 s, and 72 °C for 45 s; with a final extension at 72 °C for 5 min. Successful amplification was verified by agarose gel electrophoresis on a subset of samples in each batch.

#### 2.4.3 Library construction and sequencing

DNA libraries were constructed following the instructions of the VAHTS DNA Clean Beads (Vazyme, Nanjing, China) and Hieff NGS^®^ OnePot II DNA Library Prep Kit for MGI (Yeason BioTech, Shanghai, China). Library quality was confirmed by Agilent 2100 Bioanalyzer system (Agilent Technologies, Santa Clara, CA, USA) and the Qubit dsDNA HS Assay Kit (Thermo Fisher Scientific, Waltham, MA, USA). Sequencing was performed on the DNBSEQ-G99 platform (MGI Technology, Shenzhen, China) in single-end 50 bp mode, with a target sequencing output of approximately 1.5 million reads per sample.

#### 2.4.4 Bioinformatics

Raw FASTQ files generated by the DNBSEQ-G99 platform were quality filtered and adapter pruned using Trimmomatic (v0.36) to remove adapter sequences and low-quality read sequences before downstream bioinformatics analysis. Host sequences were subtracted by mapping to the human reference genome (hs37d5) using Bowtie2 (version 2.2.6). After removal of human-derived reads, the remaining reads were taxonomically classified using Kraken2 (v2.0.7) against a custom microbial reference database derived from GenBank Release 238 (https://ftp.ncbi.nlm.nih.gov/genbank/). The database used in this study was updated in 2023. For the study-relevant mycobacterial module, an NCBI-designated representative genome was selected as the reference for each included taxon. The module contained the publicly available reference genome for the *Mycobacterium tuberculosis complex* and 132 representative genomes corresponding to 132 nontuberculous mycobacterial species. These references supported species-level identification and, where reference coverage permitted, subspecies-level typing. Reference genomes were retained only when they had valid and unambiguous NCBI taxonomic assignments. Duplicate records, records with unresolved or inconsistent taxonomic annotations, and records flagged as potentially contaminated during internal curation were excluded before database indexing.

The positive report threshold was pre-set based on the analysis validation of the tNGS detection method developed by the company and followed the reporting algorithm described in previously published literature (Qiu et al., 2026). positive report is indicated when the species-specific readings of microorganisms meet the following criteria: M*ycobacterium tuberculosis* complex ≥200, human β -herpesvirus types 6, 7 and γ -herpesvirus types 4 ≥1000, bacteria (excluding mycobacteria), fungi, viruses (excluding the above-mentioned herpesviruses) and parasites ≥30. To reduce false positive results caused by background contamination, all detected microorganisms are corrected according to the preset no-template control (NTC) standards established during the detection validation process and interpreted after comparison with the NTC. For NTM species identification, the tNGS panel covered the following clinically relevant species/subspecies: *M. avium*, *M. intracellulare*, *M. abscessus* subsp. abscessus, *M. abscessus* subsp. massiliense, *M. kansasii*, *M. fortuitum*, and *M. chelonae*, among others. The distribution of identified NTM species is shown in **Supplementary Table 5**.

#### 2.4.5 Antimicrobial resistance mutation analysis

Variant interpretation was performed using the in-house resistance database described in Section 2.3. At the variant level, detected variants were categorized as resistance-associated, not associated with resistance, or of uncertain significance. Variants of uncertain significance did not trigger a resistant call. For NTM, interpretation was additionally based on species-specific peer-reviewed evidence, including *erm(41)* polymorphisms associated with inducible macrolide resistance in the *M. abscessus* complex.

### 2.5 Therapeutic impact assessment and patient prognosis statistics

The therapeutic impact of tNGS was evaluated by retrospectively analyzing the adjustments made by clinicians to antibacterial drug treatment after receiving tNGS reports. Treatment adjustments were defined as any changes made to the antibiotic regimen within 14 days after the tNGS report and were classified as treatment escalation (initiation, intensification, or replacement of antibiotics), treatment de-escalation (discontinuation, reduction, or reduction in treatment), and maintenance of the original regimen when deemed appropriate by the clinicians.

To reduce attribution bias, all treatment adjustments were assessed by reviewing complete clinical records, including microbiological findings, antimicrobial prescriptions, and physician notes of disease course. Treatment adjustments are classified as “tNGS-dependent” only when clinical records indicate that tNGS results have a significant impact on treatment decisions, and the treatment adjustment is consistent with the pathogen and/or drug resistance mutation detected by tNGS, and there is no other concurrent microbiological evidence to provide an independent explanation. Treatment changes resulting from other factors, such as adverse drug reactions, emerging routine microbiological outcomes, disease progression, or guideline-based management, are classified as “tNGS-independent”.

Furthermore, patient outcomes were defined according to WHO treatment categories: cure (bacteriologically confirmed conversion), improvement (clinical and/or radiological evidence of improvement without bacteriological confirmation), no improvement (persistent clinical/radiological findings), deterioration (worsening clinical status requiring treatment intensification or hospitalization), or unknown status (incomplete follow-up data). These data were subsequently utilized for statistical analysis of the overall clinical trajectory following tNGS-guided initial therapeutic intervention. Outcomes were assessed at the end of standard treatment courses or at 6 months after treatment initiation, whichever came first.

### 2.6 Outcomes

The primary outcome was the diagnostic performance of tNGS in distinguishing TB from NTM, mixed infection and other types of infection, with final clinical diagnosis as the reference standard. Secondary outcomes focused on the clinical applicability of tNGS, including comparison with conventional diagnostic approaches, evaluation of drug resistance detection and assessment of therapeutic impact. These outcomes were designed to clarify the potential of tNGS to provide more timely and accurate diagnostic information as well as guide individualized therapy of TB.

### 2.7 Statistical analysis

Categorical variables were expressed as number and percentage (%). Continuous variables data were expressed as medians with interquartile ranges (IQR). Comparisons of categorical variables across groups were performed using the chi-squared test or Fisher’s exact test, as appropriate. Comparisons of continuous variables were performed using the Mann-Whitney U test (two groups) or Kruskal-Wallis test (≥3 groups). Diagnostic performance metrics for pathogen detection and drug resistance profiling, including sensitivity, specificity, positive predictive value (PPV), negative predictive value (NPV), and overall agreement, were calculated using 2×2 contingency tables, with the final clinical diagnosis as the reference standard for pathogen diagnosis, and phenotypic DST (pDST) as the reference standard for drug resistance detection. The 95% confidence intervals (CI) for proportions were calculated using the Wilson score method. The kappa coefficient was used to evaluate the agreement between diagnostic test results and reference standards. Given the retrospective nature of the study and the multiple exploratory comparisons performed, all subgroup analyses with n < 20 were explicitly presented as hypothesis-generating and interpreted with caution. P values for comparisons between small groups were reported and presented as descriptive analyses only. Statistical analyses were performed using SPSS software (version 27.0, IBM Corp., Armonk, NY, USA) and R (version 4.3.0, R Foundation for Statistical Computing, Vienna, Austria). A two-sided P value < 0.05 was considered statistically significant.

## 3 Results

### 3.1 Enrollment and baseline clinical characteristics of patients

In this study, a total of 130 patients who met the inclusion criteria were enrolled (**Figure 1**), including 80 males and 50 females (**Table 1**). The ages of patients ranged from 13 years old to 89 years old, with a median age of 55.50 [35.00;64.75] years. A total of 78.46% of patients presented with cough and 69.23% of patients presented with productive cough. For imaging features, 49.23% of patients presented with nodular and linear opacities. According to final clinical diagnosis, patients were classified into four groups: TB (n = 91), NTM (n = 18), Other (n = 17), and Mix (n = 4). Among patients with a prior TB history (34/48), the majority were in the TB group. Notably, more than half experienced cough (72/91, 79.12%) and productive cough (63/91, 69.23%), and nearly one-third exhibited cavity formation on imaging (24/91, 26.4%). Descriptive comparison of laboratory indices showed higher white blood cell (WBC), hemoglobin (HGB), erythrocyte sedimentation rate (ESR), lymphocyte (LYMPH) and neutrophil count (NEUT), with lower platelet (PLT) levels in the Mix group while other groups (TB, NTM and Other) showed slight differences in laboratory indices. However, these comparisons are descriptive given the highly imbalanced group sizes, particularly the Mix group (n=4).

**Table 1:** Baseline characteristics and test results of enrolled patients.

	Overall (n=130)	TB (n=91)	NTM (n=18)	Other (n=17)	Mix (n=4)
Gender (n, %)					
Male	80 (61.54%)	61 (67.03%)	8 (44.44%)	9 (52.94%)	2 (50.00%)
Female	50 (38.46%)	30 (32.97%)	10 (55.56%)	8 (47.06%)	2 (50.00%)
Age (years, median, IQR)					
	55.50 [35.00;64.75]	49.00 [31.50;60.50]	63.5 [60.00;69.75]	54.00 [37.00;60.00]	62.00 [57.00;70.25]
TB history (n, %)					
	48 (36.92%)	34 (37.36%)	5 (27.78%)	7 (41.18%)	2 (50.00%)
Clinical symptoms (n, %)					
Cough	102 (78.46%)	72 (79.12%)	15 (83.33%)	12 (70.59%)	3 (75.00%)
Productive cough	90 (69.23%)	63 (69.23%)	11 (61.11%)	12 (70.59%)	4 (100.00%)
Fever	30 (23.08%)	20 (21.98%)	4 (22.22%)	5 (29.41%)	1 (25.00%)
Hemoptysis	18 (13.85%)	11 (12.09%)	4 (22.22%)	3 (17.65%)	0 (0.00%)
Underlying diseases	61 (46.92%)	31 (34.07%)	11 (61.11%)	10 (58.82%)	2 (50.00%)
Imaging features (n, %)					
Cavity	29 (22.31%)	24 (26.4%)	3 (16.67%)	2 (11.76%)	0 (0.00%)
Calcification	11 (8.46%)	7 (7.69%)	2 (11.11%)	2 (11.76%)	0 (0.00%)
Pleural thickening adhesion	4 (3.08%)	3 (3.30%)	0 (0.00%)	1 (5.88%)	0 (0.00%)
Fibrosis	1 (0.77%)	0 (0.00%)	1 (5.56%)	0 (0.00%)	0 (0.00%)
Nodular and linear opacities	64 (49.23%)	38 (41.76%)	12 (66.67%)	12 (70.59%)	2 (50.00%)
Ground-glass opacity	3 (2.31%)	2 (2.20%)	0 (0.00%)	1 (5.88%)	0 (0.00%)
Pleural effusion	4 (3.08%)	4 (4.40%)	0 (0.00%)	0 (0.00%)	0 (0.00%)
Destroyed lung	2 (1.54%)	0 (0.00%)	0 (0.00%)	1 (5.88%)	1 (25.00%)
Bronchiectasis	5 (3.85%)	1 (1.10%)	2 (11.11%)	1 (5.88%)	1 (25.00%)
Laboratory index (median, IQR)					
WBC (×10^9^/L)	6.28 [4.55;7.94]	6.30 [4.50;7.77]	5.24 [4.58;8.00]	6.36 [5.04;8.30]	7.76 [5.880;8.940]
HGB (g/L)	128.00 [115.00;140.00]	128.50 [114.00;142.00]	123.00 [116.25;133.00]	124.00 [120.00;148.00]	134.00 [130.25;137.50]
PLT (×10^9^/L)	218.00 [183.00;281.00]	218.50 [185.00;288.25]	225.50 [181.75;293.25]	212.00 [174.00;265.00]	208.00 [196.25;283.00]
CRP (mg/L)	9.00 [5.94;22.70]	10.60 [6.00;24.10]	8.13 [5.80;12.00]	9.20 [6.40;14.55]	29.80 [9.05;70.00]
PCT (ng/mL)	0.04 [0.03, 0.07]	0.05 [0.04;0.08]	0.04 [0.03;0.05]	0.04 [0.02;0.08]	0.22 [0.14;0.29]
ESR (mm/h)	22.00 [12.00;44.75]	22.00 [12.00;54.00]	21.50 [12.25;33.75]	21.00 [12.00;35.00]	34.50 [18.75;54.75]
LYMPH (×10^9^/L)	1.25 [0.98;1.64]	1.22 [0.94;1.58]	1.07 [0.88;1.36]	1.39 [1.15;1.95]	1.62 [1.41;1.85]
NEUT (×10^9^/L)	4.26 [2.98;5.76]	4.19 [2.87;5.66]	3.51 [3.13;5.87]	4.41 [3.48;5.88]	5.36 [4.36;5.97]
LYMPH% (100%)	22.50 [15.40;28.10]	22.05 [14.98;28.05]	20.55 [14.08;25.10]	24.80 [17.90;30.90]	22.30 [17.88;29.78]
NEUT% (100%)	67.20 [62.00;76.10]	67.10 [61.70;76.10]	69.05 [64.43;76.70]	69.10 [61.80;75.30]	70.70 [59.40;78.73]

n, number; IQR, interquartile range; WBC, white blood cell; HGB, hemoglobin; PLT, platelet; CRP, C-reactive protein; PCT, procalcitonin; ESR, erythrocyte sedimentation rate; LYMPH, lymphocyte; NEUT, neutrophil count.

### 3.2 Comparison of overall agreement between different detection methods and clinical diagnosis under different sample types

Using final clinical diagnosis as the reference, the overall agreement of different methods across sample types were compared (**Table 2**). In sputum, tNGS showed the highest agreement, detecting 80.9% of clinically positive cases, compared with 43.2% for culture, 39.4% for Xpert, and 25.8% for AFB. tNGS correctly identified over 90% of both TB and NTM cases, outperforming all other methods. In BALF samples, tNGS demonstrated superior performance, with an overall agreement rate of 71.3%, whereas culture and Xpert yielded 53.6% and 42.1%, respectively. Although culture reached 100% agreement for NTM, its detection of TB was limited (53.5%). tNGS maintained balanced and high performance, exceeding 80% for both TB and NTM. The T-SPOT and PPD test results are only used as a reference for TB evaluation, as these immunological tests are not recommended for the diagnosis of NTM disease; therefore, their diagnostic performance in NTM has not been evaluated.

**Table 2:** Overall agreement between different detection methods and clinical diagnosis under different sample types.

Sample	Method	Overall (n, %)	TB (n, %)	NTM (n, %)	Other (n, %)	Mix (n, %)	P value
Sputum	tNGS	38 (80.9%)	26 (92.9%)	10 (90.9%)	0 (0.0%)	2 (100.0%)	<0.0001
	Culture	42 (43.2%)	35 (52.2%)	6 (42.9%)	0 (0.0%)	1 (50.0%)	0.0023
	Xpert	26 (39.4%)	25 (56.8%)	0 (0.0%)	0 (0.0%)	1 (50.0%)	0.0001
	AFB	33 (25.8%)	29 (32.2%)	3 (17.6%)	0 (0.0%)	1 (25.0%)	0.0374
BALF	tNGS	57 (71.3%)	49 (81.7%)	6 (85.7%)	0 (0.0%)	2 (100.0%)	<0.0001
	Culture	30 (53.6%)	23 (53.5%)	5 (100.0%)	0 (0.0%)	2 (100.0%)	0.0055
	Xpert	32 (42.1%)	28 (50.0%)	3 (37.5%)	0 (0.0%)	1 (50.0%)	0.0425
Other sample type^a^	tNGS	2 (66.7%)	2 (66.7%)	0 (0.0%)	0 (0.0%)	0 (0.0%)	1
	Culture	2 (50.0%)	2 (50.0%)	0 (0.0%)	0 (0.0%)	0 (0.0%)	1
	Xpert	1 (50.0%)	1 (50.0%)	0 (0.0%)	0 (0.0%)	0 (0.0%)	1
Immunological (all specimen types combined)	T-spot	50 (63.3%)	41 (82.0%)	-^b^	–	–	–
	PPD	28 (84.8%)	26 (92.9%)	–	–	–	–

Xpert, Xpert MTB/RIF; AFB, acid-fast bacilli; BALF, bronchoalveolar lavage fluid; T-SPOT, T-SPOT.TB; PPD, purified protein derivative.

^a^ Stool, purulent fluid, urine, pleural effusion, cerebrospinal fluid and pericardial effusion.

**^b^**T-SPOT and PPD are immunological assays designed for TB infection screening. Their performance metrics for NTM detection are not calculated, as these assays are not intended for NTM diagnosis. Results shown are for TB detection.

### 3.3 Diagnostic performance of different methods for TB and NTM detection

Comparison of diagnostic performance across methods for TB and NTM detection (**Tables 3**, **4**) demonstrated that tNGS provided the most comprehensive and accurate results. The mixed infection containing the target pathogen is regarded as a positive result in the corresponding analysis, and all remaining diagnostic categories are considered as negative controls. For TB, tNGS achieved a sensitivity of 84.0% (95% CI, 75.0%–90.8%), while specificity and PPV both reached 100% (95% CI, 90.3%–100.0% and 95.4%–100.0%, respectively), resulting in an overall accuracy of 88.5% (95% CI, 81.7%–93.4%). For NTM, tNGS demonstrated a sensitivity of 90.5% (95% CI, 69.6%–98.8%), specificity of 99.1% (95% CI, 95.0%–100.0%), and accuracy of 97.7% (95% CI, 93.4%–99.5%). At the species or subspecies level, *M. intracellulare* subsp. *intracellulare* and *M. colombiense* were the predominant NTM taxa. Less frequently detected taxa included *M. abscessus* subsp. *abscessus*, *M. szulgai*, and one *M. intracellulare* isolate that was not further subtyped. Detailed distributions are provided in **Supplementary Table 5**. In contrast, for the series of conventional methods, Xpert and AFB maintained high specificity for TB, yet their utility for NTM was minimal, with sensitivity below 20% and PPV below 10%. Immunological assays such as T-SPOT and PPD showed acceptable sensitivity for TB but are not designed to diagnose NTM disease, and their specificity and accuracy for NTM were therefore not evaluated. Notably, culture, the only conventional test capable of species differentiation, exhibited high specificity for both TB and NTM but substantially lower sensitivity (56.4% and 42.9%, respectively).

**Table 3:** Diagnostic performance of different detection methods for TB.

Method	Sensitivity % (95% CI)	Specificity % (95% CI)	PPV % (95% CI)	NPV % (95% CI)	Accuracy % (95% CI)	χ²	P value	Kappa
tNGS	84.04 (75.05–90.78)	100.00 (90.26–100.00)	100.00 (95.44–100.00)	70.59 (56.17–82.51)	88.46 (81.68–93.40)	–	0.0001	0.745
Culture	56.38 (45.76–66.59)	100.00 (90.26–100.00)	100.00 (93.28–100.00)	46.75 (35.29–58.48)	68.46 (59.73–76.33)	39.024	0	0.417
Xpert	57.45 (46.82–67.59)	91.67 (77.53–98.25)	94.74 (85.38–98.90)	45.21 (33.52–57.30)	66.92 (58.13–74.92)	30.14	0	0.373
AFB	32.26 (22.93–42.75)	91.43 (76.94–98.20)	90.91 (75.67–98.08)	33.68 (24.31–44.11)	48.44 (39.52–57.43)	52.742	0	0.154
T-SPOT	82.69 (69.67–91.77)	74.07 (53.72–88.89)	86.00 (73.26–94.18)	68.97 (49.17–84.72)	79.75 (69.20–87.96)	–	0.8036	0.558
PPD	93.10 (77.23–99.15)	75.00 (19.41–99.37)	96.43 (81.65–99.91)	60.00 (14.66–94.73)	90.91 (75.67–98.08)	–	1	0.615

PPV, positive predictive value; NPV, negative predictive value.

Xpert, Xpert MTB/RIF; AFB, acid-fast bacilli; T-SPOT, T-SPOT.TB; PPD, purified protein derivative.

The null hypothesis for chi-squared tests was that sensitivity/specificity of the index test is equal to the reference standard.

**Table 4:** Diagnostic performance of different detection methods for NTM.

Method	Sensitivity % (95% CI)	Specificity % (95% CI)	PPV % (95% CI)	NPV % (95% CI)	Accuracy % (95% CI)	χ²	P value	Kappa
tNGS	90.48 (69.62–98.83)	99.08 (94.99–99.98)	95.00 (75.13–99.87)	98.18 (93.59–99.78)	97.69 (93.40–99.52)	–	1	0.913
Culture	42.86 (21.82–65.98)	98.17 (93.53–99.78)	81.82 (48.22–97.72)	89.92 (83.05–94.68)	89.23 (82.59–93.99)	–	0.0129	0.508
Xpert	19.05 (5.45–41.91)	51.38 (41.61–61.06)	7.02 (1.95–17.00)	76.71 (65.35–85.81)	46.15 (37.38–55.11)	17.5	0	-0.175
AFB	15.00 (3.21–37.89)	72.22 (62.78–80.41)	9.09 (1.92–24.33)	82.11 (72.90–89.22)	63.28 (54.31–71.62)	3.064	0.0801	-0.101

PPV, positive predictive value; NPV, negative predictive value.

Xpert, Xpert MTB/RIF; AFB, acid-fast bacilli; T-SPOT, T-SPOT.TB; PPD, purified protein derivative.

T-SPOT and PPD are not designed or validated for NTM diagnosis. Performance metrics previously reported for NTM have been removed. For completeness, their TB-specific metrics are provided in **Table 3**.

The null hypothesis for chi-squared tests was that sensitivity/specificity of the index test is equal to the reference standard.

For Xpert and AFB NTM analyses, results should be interpreted as demonstrating non-specific reactivity rather than diagnostic performance for NTM. These results are retained only to document the extent of cross-reactivity.

### 3.4 Drug resistance of tNGS

In this study, drug-resistance data from 52 patients (48 TB and 4 NTM) with available pDST results were included to evaluate drug resistance diagnostic performance of tNGS across 13 major antimycobacterial drugs (**Tables 5**, **6**). For TB, tNGS demonstrated variable sensitivity to RIF, SM, MFX and LFX (all over 50%), while specificities for all covered drugs exceeded 86% and reached 100% for INH, LFX and PAS. Among these, LFX exhibited perfect agreement across all diagnostic indicators, with 100% sensitivity, specificity, PPV, NPV and accuracy. However, the number of resistant isolates was limited for several drugs (e.g., EMB [n=9 resistant], MFX [n=4 resistant], PAS [n=1 resistant]), and sensitivity estimates for these agents should be interpreted as exploratory findings requiring validation in larger cohorts. For the four NTM cases, tNGS showed susceptibility patterns consistent with pDST in all evaluable comparisons, although the limited sample size precludes formal statistical assessment. Importantly, tNGS also provided additional resistance or susceptibility information for MFX, AZM, CLR and LZD.

**Table 5:** Diagnostic performance of tNGS for drug resistance of TB.

Drugs	pDST resistance				pDST susceptible			Sensitivity % (95% CI)	Specificity % (95% CI)	PPV % (95% CI)	NPV % (95% CI)	Accuracy % (95% CI)
	tNGS (n)		Total (n)		tNGS (n)		Total (n)					
	R	S			R	S						
RIF	12	7		19	4	25	29	63.16 (38.36–83.71)	86.21 (68.34–96.11)	75.00 (47.62–92.73)	78.12 (60.03–90.72)	77.08 (62.69–87.97)
INH	11	11		22	0	25	25	50.00 (28.22–71.78)	100.00 (86.28–100.00)	100.00 (71.51–100.00)	69.44 (51.89–83.65)	76.60 (61.97–87.70)
EMB	2	7		9	1	34	35	22.22 (2.81–60.01)	97.14 (85.08–99.93)	66.67 (9.43–99.16)	82.93 (67.94–92.85)	81.82 (67.29–91.81)
SM	8	3		11	1	32	33	72.73 (39.03–93.98)	96.97 (84.24–99.92)	88.89 (51.75–99.72)	91.43 (76.94–98.20)	90.91 (78.33–97.47)
KAN	–	–		–	1	38	39	–	97.44 (86.52–99.94)	–	–	–
AMK	–	–		–	1	39	40	–	97.50 (86.84–99.94)	–	–	–
CLR	–	–		–	1	38	39	–	97.44 (86.52–99.94)	–	–	–
MFX	3	1		4	3	33	36	75.00 (19.41–99.37)	91.67 (77.53–98.25)	50.00 (11.81–88.19)	97.06 (84.67–99.93)	90.00 (76.34–97.21)
LFX	6	0		6	0	32	32	100.00 (54.07–100.00)	100.00 (89.11–100.00)	100.00 (54.07–100.00)	100.00 (89.11–100.00)	100.00 (90.75–100.00)
PTH	–	–		–	2	19	21	–	90.48 (69.62–98.83)	–	–	–
PAS	0	1		1	0	21	21	0.00 (0.00–97.50)	100.00 (83.89–100.00)	–	95.45 (77.16–99.88)	95.45 (77.16–99.88)
RFB	–	–		12	–	–	–	–	–	–	–	–
Pa	–	–		2	–	–	–	–	–	–	–	–

RIF, rifampicin; INH, isoniazid; EMB, ethambutol; SM, streptomycin; KAN, kanamycin; AMK, amikacin; CLR, clarithromycin; MFX, moxifloxacin; LFX, levofloxacin; PTH, prothionamide; PAS, p-aminosalicylic acid; RFB, rifabutin; Pa, pasiniazid.

Sensitivity and PPV were not calculated when no resistant isolates were available for a given drug.

Due to the small number of resistant isolates for several drugs, sensitivity estimates should be interpreted with caution and are considered exploratory. Specificity estimates, derived from larger denominators, are more stable.

**Table 6:** Diagnostic performance comparison of pDST and tNGS for drug resistance of NTM.

	RIF		EMB		AMK		MFX		AZM		CLR		LZD	
	pDST	tNGS	pDST	tNGS	pDST	tNGS	pDST	tNGS	pDST	tNGS	pDST	tNGS	pDST	tNGS
No.1	I	S	R	S	S	S	–	S	–	R	–	R	–	S
No.2	S	S	I	S	S	S	S	S	–	S	–	S	–	S
No.3	S	S	R	S	S	S	S	S	–	S	–	S	–	S
No.4	S	S	I	S	S	S	S	S	I	S	–	S	I	S

R, resistant; I, intermediate; S, susceptible; RIF, rifampicin; EMB, ethambutol; AMK, amikacin; MFX, moxifloxacin; AZM, azithromycin; CLR, clarithromycin; LZD, linezolid.

NTM pDST was performed for selected drugs only, and the small NTM sample size (n=4) precludes formal statistical comparison; these results are presented descriptively only.

**Figure 2:**
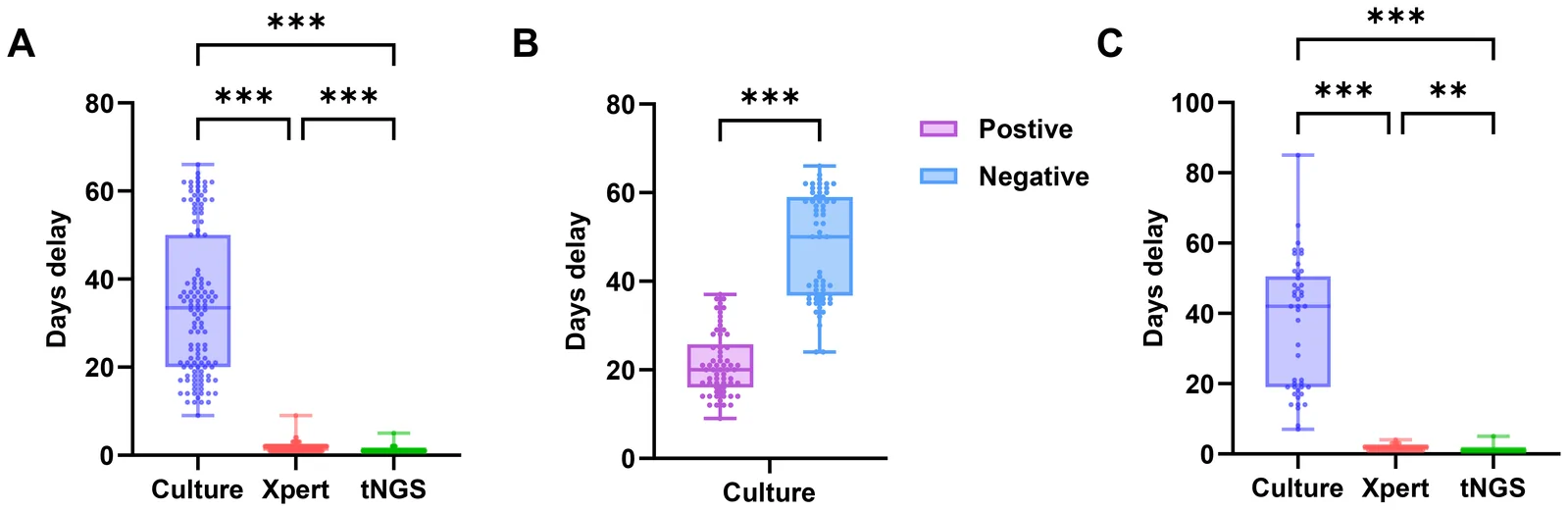
The detection time of different diagnostic methods. **(A)** Time delay in the pathogen diagnosis via culture, Xpert and tNGS. **(B)** Timeliness of culture positive and negative samples. **(C)** Time delay in the drug resistance diagnosis via culture, Xpert and tNGS. **P < 0.01, ***P < 0.001.

To evaluate the operational efficiency of different diagnostic methods, the turnaround time (TAT) was also compared (**Figure 2**). Although culture remains the reference gold standard, the average time required for pathogen identification is 34.5 days, and the phenotypic drug sensitivity test takes 36.2 days (P < 0.001; **Figures 2A, C**. The time required for the final report of culture-negative samples is longer, usually exceeding 50 days (**Figure 2B**). In contrast, tNGS can complete the comprehensive identification of pathogens and drug resistance analysis within 24 hours after sample receipt, significantly shortening the diagnostic process and facilitating the early decision-making for treatment.

Further, 37 samples with RIF-resistance data were available across culture, Xpert and tNGS (**Table 7**). tNGS showed higher specificity of 81.8% (95% CI, 59.7%–94.8%) and PPV of 73.3% (95% CI, 44.9%–92.2%) compared with Xpert, with slightly lower sensitivity (73.3% [95% CI, 44.9%–92.2%] vs. 86.7% [95% CI, 59.5%–98.3%]) but comparable accuracy of 78.4% (95% CI, 61.8%–90.2%). Both methods achieved NPV values above 80%, indicating acceptable performance. The discordant cases between Xpert and tNGS for RIF resistance (n=4) were adjudicated by reviewing pDST results and sequencing coverage depth. In two cases, tNGS detected mutations outside the Xpert target region (codon 511 and 533 of the *rpoB* gene), which were phenotypically confirmed as RIF-resistant. In the remaining two cases, Xpert detected resistance but tNGS reported susceptibility; both were phenotypically susceptible, suggesting possible Xpert false-positive calls or heteroresistance below the tNGS detection threshold.

**Table 7:** Diagnostic performance comparison of Xpert and tNGS for rifampicin resistance.

Drugs	pDST resisdance			pDST susceptible			Sensitivity % (95% CI)	Specificity % (95% CI)	PPV % (95% CI)	NPV % (95% CI)	Accuracy % (95% CI)
	gDST (n)		Total (n)	gDST (n)		Total (n)					
	R	S		R	S						
Xpert	13	2	15	6	16	22	86.67 (59.54–98.34)	72.73 (49.78–89.27)	68.42 (43.45–87.42)	88.89 (65.29–98.62)	78.38 (61.79–90.17)
tNGS	11	4	15	4	18	22	73.33 (44.90–92.21)	81.82 (59.72–94.81)	73.33 (44.90–92.21)	81.82 (59.72–94.81)	78.38 (61.79–90.17)

pDST, phenotypic drug susceptibility testing; gDST, genotypic drug susceptibility testing; Xpert, Xpert MTB/RIF; R, resistant; S, susceptible; PPV, positive predictive value; NPV, negative predictive value; RIF, rifampicin.

The Xpert MTB/RIF assay targets the 81-bp rifampicin resistance-determining region (RRDR) of the *rpoB* gene (codons 507–533). The tNGS assay sequenced the entire *rpoB* gene, including the RRDR and flanking regions.

### 3.5 Analysis of drug resistance mutations by tNGS

**Figure 3:**
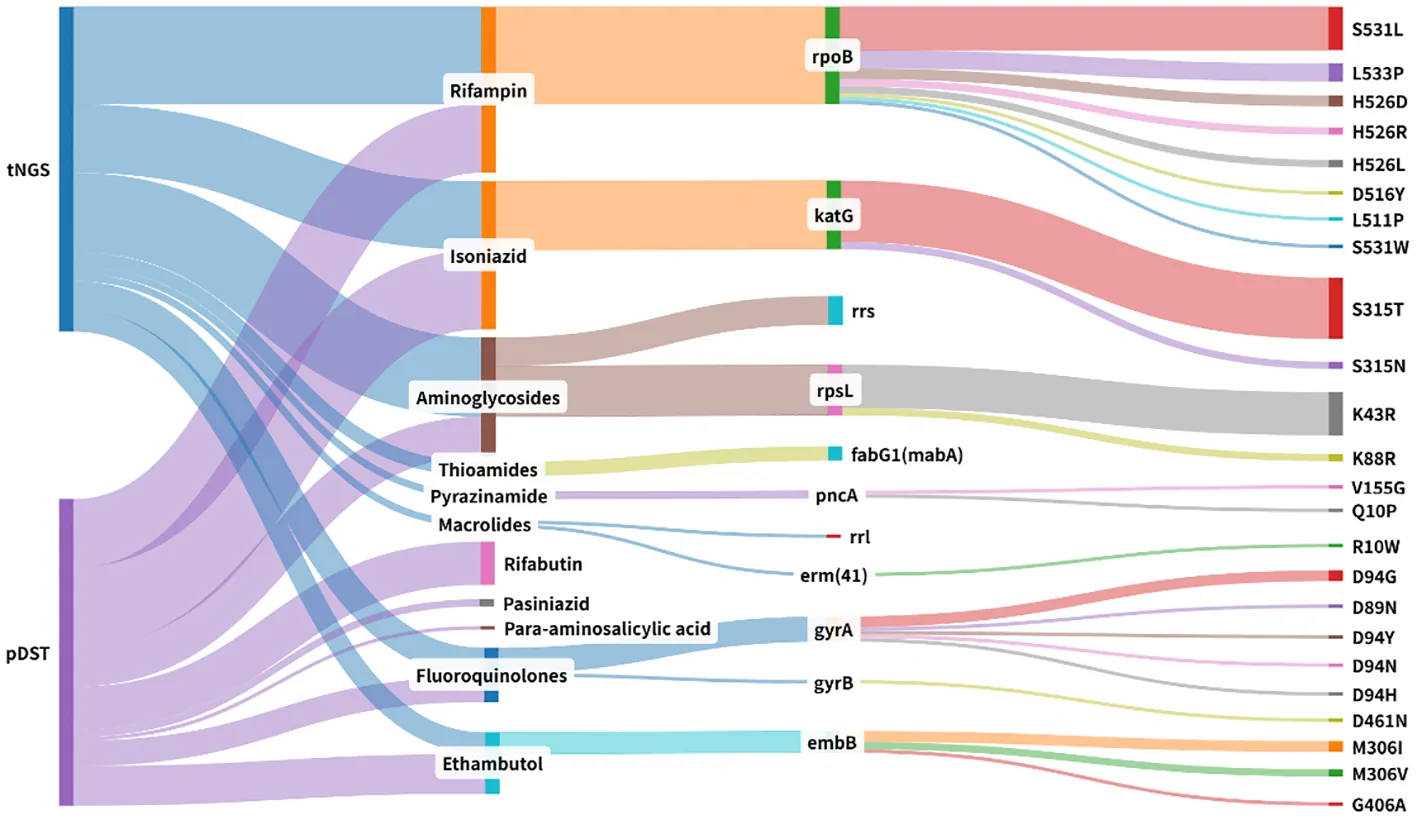
Analysis of TB drug resistance sites detected by tNGS.

tNGS enables simultaneous identification of resistance-associated genes and mutations across multiple anti-tuberculosis drugs, effectively overcoming the limitations of pDST. In this study, resistance-associated mutations were detected in 40 of 130 samples (**Figure 3**). RIF resistance was mainly attributed to mutations within the *rpoB* gene, with Ser531Leu (S531L) (n=12), Leu533Pro (L533P) (n=5) and His526Asp/Leu/Arg (H526D/L/R) (n=5) detected. INH resistance occurred in 19 cases, all involving *katG* mutations, primarily Ser315Thr (n = 17) and occasionally Ser315Asn (S315N) (n=2). Aminoglycoside resistance (including SM, KAN, and AMK) was associated with *rpsL* mutations such as Lys43Arg (K43R) (n=12) and Lys88Arg (K88R) (n=2), as well as *rrs* mutations identified in 8 cases. Fluoroquinolone resistance involved 8 cases with mutations in *gyrA*(Asp94Gly/Tyr/Asn/His [D94G/Y/N/H] and Asp89Asn [D89N]) or *gyrB*(Asp461Asn [D461N]). EMB resistance was linked to *embB* mutations (Met306Ile/Val [M306I/V] and Gly406Ala [G406A]) in 6 samples. Mutations related to thioamide resistance (PTH) were identified in 4 samples, primarily within fabG1. Two PZA-resistant cases showed *pncA* mutations (Val155Gly [V155G] and Gln10Pro [Q10P]). Additionally, macrolide resistance among NTM isolates was linked to mutations in *erm(41)* and *rrl*. The samples can be further classified according to drug resistance type into RR-TB (n=27), MDR-TB (n=16), and XDR-TB (n=6), underscoring the ability of tNGS to provide comprehensive genotypic resistance insights in a single assay. Uncommon mutations were also identified, including *rpoB* Leu511Pro (L511P) and S531W, *katG* S315N, *gyrA* D89N, and *pncA* V155G and Q10P. Although these mutations are less prevalent than canonical hotspots, accumulating evidence indicates their clinical relevance in conferring phenotypic resistance or reduced drug susceptibility. Such mutations are likely missed by conventional molecular assays such as Xpert, which is largely restricted to common *rpoB* mutations, whereas pDST does not provide genotypic information and is limited by prolonged turnaround times.

### 3.6 Therapeutic impact assessment and patient prognosis statistics

**Figure 4:**
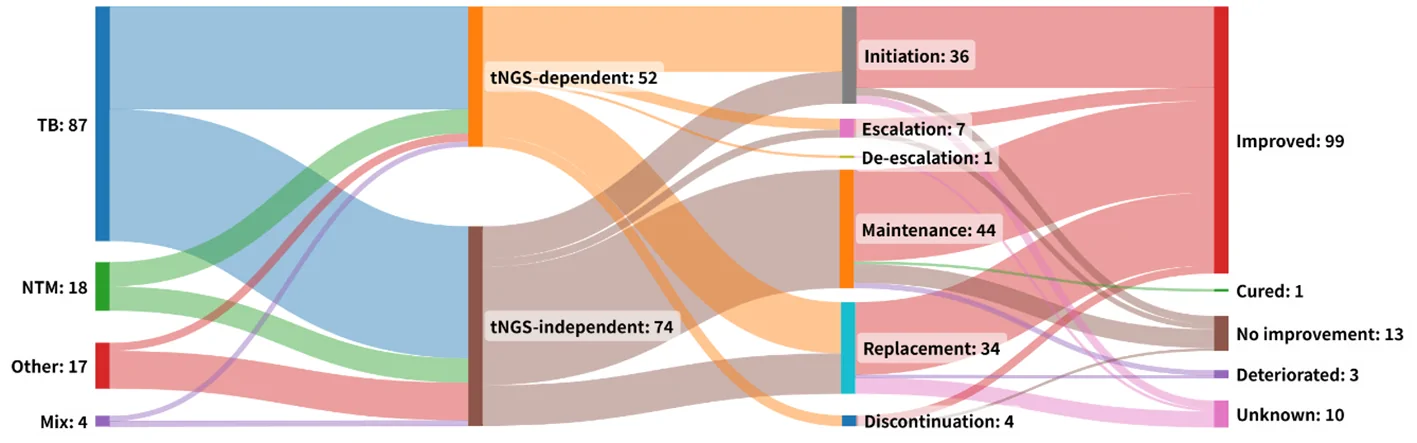
Sankey diagram of patient drug adjustment and outcomes.

The treatment efficacy and patient outcomes were evaluated in 126 patients with complete follow-up data (**Figure 4**; **Supplementary Table 6**). After obtaining the tNGS test results, a total of 52 patients (41.3%) adjusted their antibiotic treatment regimens, including 38 patients in the TB group (43.7% of the 87 traceable follow-up cases), 9 patients in the NTM group (50.0% of the 18 cases), 3 patients in the Other group (17.6% of the 17 cases), and 2 patients in the Mix group (50.0% of the 4 cases). Treatment adjustments include increasing treatment intensity or initiating treatment (n=28), reducing treatment intensity or discontinuing medication (n=5), and changing treatment regimens (that is, replacing one or more antibacterial drugs without altering the overall treatment intensity; n=19).

Among the 52 patients who received treatment adjustments after the tNGS report, 43 (82.7%) improved clinical symptoms, 6 had no improvement or deterioration of the condition, and the outcome data of 3 patients was unknown or missing. Among the 74 patients who did not undergo treatment adjustments after the tNGS report, 1 was diagnosed as cured, 56 had improved clinical symptoms, 10 had no improvement or deterioration of the condition, and the outcome data of 7 patients were unknown or missing (**Supplementary Table 5**).

## 4 Discussion

This study comprehensively evaluated the diagnostic performance of tNGS in TB and related mycobacterial infections. The results showed that tNGS could rapidly and accurately distinguish TB from clinically relevant NTM infections, was applicable to different types of samples, and achieved species-level identification of the main NTM pathogens detected in the study cohort. Additionally, tNGS simultaneously characterized the drug resistance-related mutations at key mycobacterial drug targets, providing clinically significant information for the selection of antibacterial drugs and treatment adjustments. These findings support the potential value of tNGS as a rapid and comprehensive diagnostic method for mycobacterial infections and an auxiliary tool for individualized clinical management.

In clinical practice, the limitations of TB diagnosis largely arise from difficulties in differentiating TB from NTM, identifying mixed infections, and distinguishing TB from other bacterial infections with overlapping clinical and radiological features (Liang et al., 2024; Feng et al., 2025). Epidemiological studies have shown that approximately 6.3% of patients initially suspected of TB are ultimately diagnosed with NTM infection (Yu et al., 2016). In high-burden regions such as Shanghai and Beijing, NTM accounts for 15–40% of culture-positive cases, with a steadily increasing trend in recent years (Wu et al., 2014; Huang et al., 2020). These factors substantially complicate early and accurate diagnosis, particularly in settings with a high prevalence of mycobacterial diseases (Cai et al., 2022; Wang et al., 2024; Xu et al., 2024). Our cohort demonstrated significant clinical and radiological overlap between TB, NTM, and mixed infections, with more than half of the patients across all groups presenting with similar symptoms such as cough, phlegm, and nodular and linear opacities on chest imaging.

Conventional diagnostic methods mainly target TB and struggle to identify co-infection of TB and NTM, increasing the risk of diagnostic confusion and inappropriate treatment (Dong et al., 2025). In contrast, tNGS has emerged as a powerful tool in infectious disease diagnostics due to its capacity for broad-spectrum, parallel pathogen detection (Wang J. et al., 2025). Our results demonstrated that tNGS could provide a clear diagnostic advantage by enabling concurrent detection and differentiation of TB, NTM, mixed along with bacterial infections in a single assay. Using the final clinical diagnosis as the evaluation criterion, in sputum samples, tNGS achieved positive detection rates of 92.9%, 90.9%, and 100% for TB, NTM, and mixed infections, respectively; while in BALF samples, the corresponding positive detection rates were 81.7%, 85.7%, and 100%. Furthermore, tNGS showed high sensitivity and specificity for both TB (84.0% sensitivity and 100.0% specificity) and NTM (90.5% sensitivity and 99.1% specificity), with strong agreement with final clinical diagnoses, while also identifying cases of co-infection missed by conventional methods. Given that misdiagnosis or missed diagnosis of TB could lead to delayed or inappropriate therapy (Liang et al., 2024), our findings align with previous reports supporting the robust performance of tNGS in mycobacterial detection and reinforce its clinical value as a comprehensive diagnostic tool in complex infectious scenarios. As shown in the results and **Supplementary Table 5**, tNGS was able to perform species-level identification of the clinically relevant multidrug-resistant NTM pathogens detected in this study cohort. The predominant species were *M. intracellulare* and *M. colombiense*. This taxonomic resolution is of great clinical significance because there are significant differences in the antimicrobial drug sensitivity and treatment recommendations among different NTM species and subspecies. For example, distinguishing *M. abscessus* subsp. *abscessus* from subsp. *massiliense* is particularly important, as the former carries the functional *erm(41)* gene that can induce macrolide resistance, while the latter typically lacks this resistance mechanism, thus resulting in different treatment strategies.

Additionally, the increasing global burden of RR-TB, MDR-TB, and XDR-TB poses substantial clinical challenges, given their association with prolonged treatment courses, limited therapeutic options, higher healthcare costs, and increased mortality (Trajman et al., 2025). tNGS offers a major advantage by providing genotypic resistance profiling, enabling rapid characterization of resistance patterns to inform personalized treatment strategies (Murphy et al., 2023; Colman et al., 2025; Xu et al., 2025). In this study, using pDST as the reference, tNGS showed overall favorable diagnostic performance across multiple antimycobacterial drugs. A total of 27 distinct resistance-associated genotypes were identified in 40 samples within approximately 24 hours from sample receipt, covering resistance to RIF, INH, aminoglycosides, fluoroquinolones, EMB, thioamides, pyrazinamide, and macrolides. This extensive coverage underscores the ability of tNGS to capture complex and heterogeneous resistance landscapes in a single assay, providing a resistance profile that more closely reflects the clinical complexity of drug-resistant TB (Schwab et al., 2024; Ou et al., 2025). However, it is important to acknowledge the limitations of our drug resistance analysis. For several drugs, the number of phenotypically resistant isolates was small (e.g., EMB [n=9], MFX [n=4], PAS [n=1]), limiting the stability and reliability of sensitivity estimates. Sensitivity calculated from a small denominator is inherently imprecise and should be interpreted as an exploratory finding, whereas specificity, derived from larger numbers of susceptible isolates, provides more robust estimates. These results require validation in larger, multicenter cohorts with greater numbers of resistant isolates across all antimycobacterial agents.

Beyond common resistance-conferring mutations, tNGS also demonstrated a clear advantage in detecting less frequent and atypical resistance-associated variants (Wu et al., 2022). In our cohort, uncommon mutations were identified, including *rpoB*L533P, L511P and S531W associated with RIF resistance, *katG*S315N with INH resistance, *gyrA*D89N with fluoroquinolone resistance, and *pncA*V155G and Q10P linked to pyrazinamide resistance. Although these mutations are less prevalent than canonical hotspots, accumulating evidence indicates that they are clinically relevant and can confer phenotypic resistance or contribute to reduced drug susceptibility (Hauck et al., 2009; Malik et al., 2012; Jo et al., 2017). Such mutations are likely to be missed by conventional molecular assays, particularly Xpert, which is largely restricted to common *rpoB* mutations associated with RIF resistance. In contrast, pDST does not provide genotypic information and is inherently limited by prolonged turnaround times and potential culture failure (Nie et al., 2023; Rigouts et al., 2023).

By leveraging broad target coverage, tNGS enables comprehensive mutation detection and delivers actionable resistance information within 1–2 days, substantially shortening the diagnostic window (Colman et al., 2025). This advantage is particularly relevant for NTM infections, as culture-based identification and susceptibility testing often require several weeks because of slow growth rates and complex laboratory workflows. In contrast, tNGS allows rapid species-level identification of NTM directly from clinical specimens (Mohamed et al., 2021), and at the same time, our study confirmed that tNGS could detect resistance genes associated with macrolides, fluoroquinolones, aminoglycosides, and other drugs commonly used in the treatment of NTM. Such early access to both etiological and resistance information is clinically meaningful, as delayed or empiric therapy for NTM may lead to prolonged disease courses or inappropriate antimicrobial exposure.

From a clinical perspective, timely and comprehensive resistance profiling provides an important reference for treatment decision-making. In this study, treatment regimens were adjusted in 52 cases with reference to tNGS results, including escalation, de-escalation, or modification of antimicrobial therapy. Subsequent follow-up showed that 82.7% of these patients experienced clinical improvement. These observations suggest that tNGS-derived information was integrated into clinical decision-making in a substantial proportion of cases. However, the observed improvement rates should be interpreted descriptively. The study design does not permit attribution of improved outcomes specifically to tNGS-guided therapy, as multiple confounders—including baseline severity, underlying diagnosis, and concurrent supportive care—may influence clinical trajectories. Future prospective studies with predefined treatment protocols and systematic outcome ascertainment will be necessary to rigorously evaluate the impact of tNGS-guided therapy.

Our findings should be evaluated within the context of the broader literature on sequencing-based diagnostics for mycobacterial infections. Recent studies have evaluated various genomic approaches, including whole-genome sequencing, metagenomic sequencing, and targeted sequencing platforms. For instance, a study using nanopore sequencing to identify mycobacterial species and resistance-associated mutations reported promising turnaround times and the advantage of long-read sequencing for resolving complex genomic regions (Carandang et al., 2025). The present study complements such work by demonstrating the clinical utility of an alternative, amplification-based tNGS approach on a short-read sequencing platform, which may offer advantages in diagnostic laboratory settings including higher throughput and established clinical validation pipelines. This study supplemented the work by adopting an amplification-based tNGS alternative method on a short-read long sequencing platform, demonstrating the application value of this method in clinical settings. This method may offer advantages in diagnostic laboratory environments, such as higher throughput and a mature clinical validation process. However, when comparing different sequencing platforms, in addition to practical factors such as laboratory infrastructure, workflow, and cost, platform-specific technical characteristics should also be considered, including read length, sequencing error characteristics, sequencing depth, and variant detection performance. These factors may all affect diagnostic accuracy and the detection effectiveness of drug resistance mutations. Therefore, the selection of sequencing technology should be guided by specific clinical applications and diagnostic goals, rather than solely based on operational considerations. Other recent studies have similarly demonstrated the feasibility and accuracy of tNGS for MTB detection and resistance profiling in diverse clinical settings (Colman et al., 2025; Gou et al., 2025; Wang M. et al., 2025), supporting the growing evidence base for sequencing-driven diagnostics in mycobacterial disease management.

An important consideration for clinical practice is how tNGS should be positioned relative to mycobacterial culture. Although tNGS offers substantial advantages in speed and breadth of resistance detection, it does not replace mycobacterial culture. Culture remains essential for recovering viable isolates, enabling complete phenotypic drug susceptibility testing (particularly for drugs without well-characterized genotypic resistance mechanisms), and facilitating public health investigations through molecular epidemiological typing and transmission studies. Additionally, culture provides a necessary backup for cases where tNGS results are equivocal or when phenotypic-genotypic discordance requires resolution. We therefore envision tNGS as a complementary diagnostic tool that augments, rather than replaces, conventional culture-based methods in the clinical mycobacteriology laboratory. Therefore, we believe that tNGS as a diagnostic tool can enhance traditional culture methods in clinical TB laboratories and can be used as a powerful supplement to traditional culture methods for the diagnosis of TB infection. However, prospective evaluation is still needed to effectively integrate tNGS into the clinical diagnostic process.

While the diagnostic advantages of tNGS are clearly demonstrated, several practical considerations influence its implementation in routine clinical laboratories. The cost of tNGS, including instrumentation (sequencing platform, automated library preparation systems), consumables (library preparation kits, flow cells), and bioinformatics infrastructure (computing resources, curated databases), remains substantially higher than that of conventional methods such as Xpert or culture. Specialized bioinformatics expertise is required for data analysis, variant interpretation, and database curation, which may not be readily available in all settings. Furthermore, the workflow demands rigorous quality control procedures to prevent cross-contamination and ensure reproducible results. However, as sequencing costs continue to decline and automated, user-friendly bioinformatics pipelines become more widely available, the feasibility of integrating tNGS into routine diagnostic workflows is likely to improve. Centralized or reference laboratory models, with sample referral networks, may represent an intermediate implementation strategy for resource-limited settings.

Despite advantages, our research still has several limitations. First, the sample size within certain subgroups remains limited, especially for less common categories such as NTM (n=18) and mixed infection (n=4), which restricts the robustness of subgroup analysis. The small number of cases in these categories contributes to wide confidence intervals around performance estimates and limits the generalizability of our findings. These analyses should be considered exploratory and hypothesis-generating. Second, not all specimens were tested for pDST, and the relatively small number of specimens with complete resistance data may lead to imprecise sensitivity estimates for several drugs. For agents with fewer than 10 resistant isolates, sensitivity estimates should be interpreted with particular caution. Third, although clinical follow-up was performed, not all patients had complete outcome data and long-term prognosis was not systematically assessed. Given the chronic and recurrent nature of TB, extended follow-up would provide stronger evidence of the impact of tNGS-dependent therapy on patient outcomes. Fourth, the heterogeneity of composition of mixed infections we included limited the in-depth assessment of the performance of tNGS in this setting. Fifth, the retrospective, observational design precludes causal inference regarding the impact of tNGS on clinical outcomes. The observed associations may be confounded by unmeasured variables, including baseline severity, clinician experience, and concurrent interventions. Finally, the study included diverse specimen types (sputum, BALF, and various non-respiratory samples). Although we provided stratified analyses by specimen category, the heterogeneity of sample matrices may have influenced overall performance estimates. Future studies should evaluate performance in larger, specimen-type-specific cohorts. Overall, these limitations highlight the need for future multicenter studies with larger cohorts, comprehensive resistance testing and long-term follow-up to establish a more complete evaluation framework for tNGS in TB and NTM management.

## 5 Conclusions

In conclusion, tNGS enables rapid and accurate differentiation between TB and NTM and provides reliable drug resistance profiling with actionable mutation site information. Our findings highlight its potential to guide timely and optimized treatment adjustment, underscoring the clinical utility of tNGS as a complementary tool in the management of mycobacterial infections. The integration of tNGS into routine diagnostic methods, may improve the speed and precision of clinical decision-making for patients with suspected mycobacterial disease. However, larger prospective studies with systematic follow-up are warranted to definitively establish the clinical impact of tNGS-guided therapy on patient outcomes.

## Data Availability

The original contributions presented in the study are included in the article/**Supplementary Material**. Further inquiries can be directed to the corresponding author.

## References

[B1] Akalu T. Y. Clements A. C. A. Gebreyohannes E. A. Xu Z. Bai L. Alene K. A. (2024). Risk factors for diagnosis and treatment delay among patients with multidrug-resistant tuberculosis in Hunan Province, China. BMC Infect. Dis. 24, 159. doi: 10.1186/s12879-024-09036-2 38308252 PMC10835895

[B2] Cai J. L. Shuai C. Wei P. Yue H. C. Li L. J. Qi J. (2022). Increasing trends and species diversity of nontuberculous mycobacteria in a coastal migrant city—Shenzhen, China. Biomed. Environ. Sci. 35, 146. 35197180 10.3967/bes2022.020

[B3] Carandang T. H. D. C. Cunanan D. J. Co G. S. Pilapil J. D, Garcia J. I. Restrepo B. I. . (2025). Diagnostic accuracy of nanopore sequencing for detecting Mycobacterium tuberculosis and drug-resistant strains: a systematic review and meta-analysis. Sci. Rep. 15, 11626. doi: 10.1038/s41598-025-90089-x 40185766 PMC11971303

[B4] Chen H. Bi H. Li P. Yang Z. Wen L. Sun B. . (2025). Evaluation of pulmonary tuberculosis disease burden in Shenyang, China, 2023. BMC Infect. Dis. 25, 166. doi: 10.1186/s12879-025-10572-8 39905313 PMC11796274

[B5] Chen Y. Fan L. Ren Z. Yu Y. Sun J. Wang M. . (2025a). Sensitive diagnosis of paucibacillary tuberculosis with targeted next-generation sequencing: a molecular diagnostic study. BMC Med. 23, 178. doi: 10.1186/s12916-025-03996-1 40140883 PMC11948806

[B6] Chen Y. Tang H. Zheng J. Yang Q. Han D. (2025b). Transforming tuberculosis diagnosis with clinical metagenomics: progress and roadblocks. J. Clin. Microbiol. 63, e00537-25. doi: 10.1128/jcm.00537-25 40970697 PMC12710363

[B7] Colman R. E. Seifert M. De la Rossa A. Georghiou S. B. Hoogland C. Uplekar S. . (2025). Evaluating culture-free targeted next-generation sequencing for diagnosing drug-resistant tuberculosis: a multicentre clinical study of two end-to-end commercial workflows. Lancet Infect. Dis. 25, 325–334. doi: 10.1016/s1473-3099(24)00586-3 39486428 PMC11871994

[B8] Deng L. Zhao F. Li Z. Zhang W. He G. Ren X. (2024). Epidemiological characteristics of tuberculosis incidence and its macro-influence factors in Chinese mainland during 2014–2021. Infect. Dis. Poverty 13, 34. doi: 10.1186/s40249-024-01203-6 38773558 PMC11107005

[B9] Dheda K. Mirzayev F. Cirillo D. M. Udwadia Z. Dooley K. E. Chang K. C. . (2024). Multidrug-resistant tuberculosis. Nat. Rev. Dis. Primers 10, 22. doi: 10.1136/thorax.58.4.347 38523140 PMC13335523

[B10] Dong Q. Wang M. Wang Z. Shi J. Xie J. Li X. . (2025). Extrapulmonary comparisons between Mycobacterium tuberculosis and non-tuberculous mycobacteria: From manifestations and diagnosis to treatment. Infect. Drug Resist. 18, 2613–2627. doi: 10.2147/idr.s515196 40432812 PMC12107284

[B11] Eigner U. Köffer J. Betz U. Koglin J. Richter E. (2025). Evaluation of the cobas MTB and MTB-RIF/INH assay in clinical samples for the detection of Mycobacterium tuberculosis in respiratory specimens. J. Clin. Microbiol. 63, e0195924. doi: 10.1128/jcm.01959-24 40130921 PMC12077164

[B12] Farhat M. Cox H. Ghanem M. Denkinger C. M. Rodrigues C. Abd El Aziz M. S. . (2024). Drug-resistant tuberculosis: a persistent global health concern. Nat. Rev. Microbiol. 22, 617–635. doi: 10.1038/s41579-024-01025-1 38519618

[B13] Feng Y. Guo J. Luo S. Zhang Z. (2025). Phenotyping nontuberculous mycobacterial lung disease: Comparative analysis of clinical and imaging features in a TB-endemic setting. Infect. Drug Resist. 18, 3527–3534. doi: 10.2147/idr.s529466 40689221 PMC12273711

[B14] Gou B. Zhang C. Song Z. Huang Z. Lu S. (2025). Targeted next-generation sequencing for rapid tuberculosis detection: a systematic review and meta-analysis. Clin. Chim. Acta 577, 120469. doi: 10.1016/j.cca.2025.120469 40633710

[B15] Hauck Y. Fabre M. Vergnaud G. Soler C. Pourcel C. (2009). Comparison of two commercial assays for the characterization of rpoB mutations in Mycobacterium tuberculosis and description of new mutations conferring weak resistance to rifampicin. J. Antimicrob. Chemother. 64, 259–262. doi: 10.1093/jac/dkp204 19520715

[B16] Huang J. Li Y. Zhao Y. Yang W. Xiao M. Kudinha T. . (2020). Prevalence of nontuberculous mycobacteria in a tertiary hospital in Beijing, China, January 2013 to December 2018. BMC Microbiol. 20, 158. doi: 10.1186/s12866-020-01840-5 32532202 PMC7291475

[B17] Huh H. J. Song D. J. Ki C. S. Lee N. Y. (2019). Is cross-reactivity with nontuberculous mycobacteria a systematic problem in the Xpert MTB/RIF assay? Tuberc Respir. Dis. (Seoul) 82, 88–89. doi: 10.4046/trd.2018.0075 30574692 PMC6304324

[B18] Jo K. W. Lee S. Kang M. R. Sung H. Kim M. N. Shim T. S. (2017). Frequency and type of disputed rpoB mutations in Mycobacterium tuberculosis isolates from South Korea. Tuberc Respir. Dis. (Seoul) 80, 270–276. doi: 10.4046/trd.2017.80.3.270 28747960 PMC5526954

[B19] Liang Q. Jiang X. Jia J. Zhao L. Li Y. Wang F. . (2024). An early and trustable indicator suggestive of non-tuberculosis mycobacteria isolation in a high tuberculosis burden setting. J. Infection 88, 106149. doi: 10.1016/j.jinf.2024.106149 38574774

[B20] Malik S. Willby M. Sikes D. Tsodikov O. V. Posey J. E. (2012). New insights into fluoroquinolone resistance in Mycobacterium tuberculosis: Functional genetic analysis of gyrA and gyrB mutations. PloS One 7, e39754. doi: 10.1002/9781119593522.ch6 22761889 PMC3386181

[B21] Meehan C. J. Goig G. A. Kohl T. A. Verboven L. Dippenaar A. Ezewudo M. . (2019). Whole genome sequencing of Mycobacterium tuberculosis: current standards and open issues. Nat. Rev. Microbiol. 17, 533–545. doi: 10.1038/s41598-023-38384-3 31209399

[B22] Mohamed S. Köser C. U. Salfinger M. Sougakoff W. Heysell S. K. (2021). Targeted next-generation sequencing: a Swiss army knife for mycobacterial diagnostics? Eur. Respir. J. 57, 2004077. doi: 10.1183/13993003.04077-2020 33737379 PMC8920038

[B23] Murphy S. G. Smith C. Lapierre P. Shea J. Patel K. Halse T. A. . (2023). Direct detection of drug-resistant Mycobacterium tuberculosis using targeted next generation sequencing. Front. Public Health 11, 1206056. doi: 10.3389/fpubh.2023.1206056 37457262 PMC10340549

[B24] Naidoo K. Perumal R. Ngema S. L. Shunmugam L. Somboro A. M. (2024). Rapid diagnosis of drug-resistant tuberculosis—opportunities and challenges. Pathogens 13, 27. doi: 10.3390/pathogens13010027 38251335 PMC10819693

[B25] Nie Q. Sun D. Zhu M. Tu S. Chen N. Chen H. . (2023). Phenotypic drug susceptibility characterization and clinical outcomes of tuberculosis strains with A-probe mutation by GeneXpert MTB/RIF. BMC Infect. Dis. 23, 832. doi: 10.1186/s12879-023-08509-0 38012619 PMC10680243

[B26] Ou X. Pei S. Li H. Qin Z. Anthony R. Wang J. . (2025). A comprehensive evaluation of a novel targeted-sequencing workflow for Mycobacterium species identification and anti-tuberculosis drug-resistance detection. Front. Cell. Infect. Microbiol. 15, 1584237. doi: 10.3389/fcimb.2025.1584237 40552123 PMC12183266

[B27] Pai M. Behr M. (2016). Latent Mycobacterium tuberculosis infection and interferon-gamma release assays. Microbiol. Spectr. 4, 10.1128/microbiolspec.tbtb1122–0023-2016. doi: 10.1038/nrmicro2236-c1 27763261

[B28] Qi X. Yang Q. Cai J. Wu J. Gao Y. Ruan Q. . (2024). Transcriptional profiling of human peripheral blood mononuclear cells in household contacts of pulmonary tuberculosis patients provides insights into mechanisms of Mycobacterium tuberculosis control and elimination. Emerg. Microbes Infect. 13, 2295387. doi: 10.1080/22221751.2023.2295387 38088554 PMC10763880

[B29] Qiu D. Wang Y. Guo S. Xue T. Wang A. Chen S. . (2026). Lung puncture biopsies based targeted NGS increase clinical benefit to patients with short-term progressive pulmonary lesions suspected to be benign. Int. J. Infect. Dis. 162, 108232. doi: 10.1016/j.ijid.2025.108232 41265634

[B30] Rahman S. M. M. Nasrin R. Kabir S. Kabir F. Rahman A. M. R. Uddin M. K. M. . (2025). Performance of Xpert MTB/RIF ultra for the diagnosis of tuberculous meningitis in children using cerebrospinal fluid. Sci. Rep. 15, 13060. doi: 10.1038/s41598-025-97664-2 40240519 PMC12003733

[B31] Rigouts L. Keysers J. Rabab R. Fissette K. van Deun A. de Jong B. C. (2023). GeneXpert MTB/RIF Ultra performance to detect uncommon rpoB mutations in Mycobacterium tuberculosis. BMC Res. Notes 16, 146. doi: 10.1186/s13104-023-06394-z 37452349 PMC10347863

[B32] Saikaew S. Sangboonruang S. Pongsararuk R. Srilohasin P. Butr-Indr B. Intorasoot S. . (2025). Gold nanoparticle-enhanced recombinase polymerase amplification for rapid visual detection of Mycobacterium tuberculosis. Biosens.-Basel 15, 607. doi: 10.3390/bios15090607 41002347 PMC12467982

[B33] Schwab T. C. Perrig L. Göller P. C. Guebely De la Hoz F. F. Lahousse A. P. Minder B. . (2024). Targeted next-generation sequencing to diagnose drug-resistant tuberculosis: a systematic review and meta-analysis. Lancet Infect. Dis. 24, 1162–1176. doi: 10.1016/s1473-3099(24)00263-9 38795712 PMC11881551

[B34] Teo A. K. J. Singh S. R. Prem K. Hsu L. Y. Yi S. (2019). Delayed diagnosis and treatment of pulmonary tuberculosis in high-burden countries: a systematic review protocol. BMJ Open 9, e029807. doi: 10.1136/bmjopen-2019-029807 31289094 PMC6629411

[B35] Trajman A. Campbell J. R. Kunor T. Ruslami R. Amanullah F. Behr M. A. . (2025). Tuberculosis. Lancet 405, 850–866. doi: 10.1007/978-3-031-15955-8_27 40057344

[B36] Tran B. M. Larsson J. Grip A. Karempudi P. Elf J. (2025). Phenotypic drug susceptibility testing for Mycobacterium tuberculosis variant bovis BCG in 12 hours. Nat. Commun. 16, 4366. doi: 10.1038/s41467-025-59736-9 40348759 PMC12065818

[B37] Wang X. Li T. Liu Y. Zhu Y. Chen L. Gao Y. (2024). Clinical characteristics of patients with nontuberculous mycobacterium pulmonary disease in Fuyang, China: A retrospective study. Infection Drug Resistance 14, 3989–4000. doi: 10.2147/idr.s475652 39296777 PMC11410028

[B38] Wang J. Cheng S. Zhang Y. Qian J. Zhang Y. Zhuang X. (2025). Post-pandemic surveillance of seven key respiratory pathogens using targeted next-generation sequencing (tNGS): A regional epidemiological study from Shandong, China. Diagn Microbiol. Infect. Dis. 113, 116931. doi: 10.1016/j.diagmicrobio.2025.116931 40483887

[B39] Wang M. Wu Y. Xi Y. Fan L. Sun F. Zhou G. . (2025). Enhanced targeted NGS assay for comprehensive diagnosis in tuberculosis and drug-resistant tuberculosis patients. Int. J. Antimicrob. Agents 66, 107598. doi: 10.1016/j.ijantimicag.2025.107598 40846033

[B40] Wright P. W. Wallace R. J. Jr. Wright N. W. Brown B. A. Griffith D. E. (1998). Sensitivity of fluorochrome microscopy for detection of Mycobacterium tuberculosis versus nontuberculous mycobacteria. J. Clin. Microbiol. 36, 1046–1049. doi: 10.1128/jcm.36.4.1046-1049.1998 9542935 PMC104687

[B41] Wu S. H. Xiao Y. X. Hsiao H. C. Jou R. (2022). Development and assessment of a novel whole-gene-based targeted next-generation sequencing assay for detecting the susceptibility of Mycobacterium tuberculosis to 14 drugs. Microbiol. Spectr 10, e0260522. doi: 10.1128/spectrum.02605-22 36255328 PMC9769975

[B42] Wu J. Zhang Y. Li J. Lin S. Wang L. Jiang Y. . (2014). Increase in nontuberculous mycobacteria isolated in Shanghai, China: Results from a population-based study. PloS One 9, e109736. doi: 10.1371/journal.pone.0109736 25330201 PMC4199589

[B43] Xu Q. Chen Q. Qiu W. Liu L. Zeng W. Chen J. . (2025). Application of targeted next-generation sequencing for pathogens diagnosis and drug resistance prediction in bronchoalveolar lavage fluid of pulmonary infections. Front. Cell. Infect. Microbiol. 15, 1590881. doi: 10.3389/fcimb.2025.1590881 40552119 PMC12183283

[B44] Xu N. Li L. Wu S. (2024). Epidemiology and laboratory detection of non-tuberculous mycobacteria. Heliyon 10, e35311. doi: 10.1016/j.heliyon.2024.e35311 39166010 PMC11334812

[B45] Xu C. Zhao Y. (2025). Commit, invest and deliver: Towards achieving end tuberculosis strategy goals through active case finding and preventive treatment in China. China CDC Weekly 7, 407. 40226522 10.46234/ccdcw2025.068 PMC11986444

[B46] Yao Q. Zhou Q. H. Shen Q. L. Wang X. C. Hu X. H. (2022). Imaging characteristics of pulmonary BCG/TB infection in patients with chronic granulomatous disease. Sci. Rep. 12, 11765. doi: 10.1038/s41598-022-16021-9 35817807 PMC9273607

[B47] Yu X. Liu P. Liu G. Zhao L. Hu Y. Wei G. . (2016). The prevalence of non-tuberculous mycobacterial infections in mainland China: Systematic review and meta-analysis. J. Infect. 73, 558–567. doi: 10.1016/j.jinf.2016.08.020 27717784

[B48] Zhang H. Tang M. Li D. Xu M. Ao Y. Lin L. (2024). Applications and advances in molecular diagnostics: revolutionizing non-tuberculous mycobacteria species and subspecies identification. Front. Public Health 12. doi: 10.3389/fpubh.2024.1410672 38962772 PMC11220129

