# Supplementary material for: Clinical utility of targeted next-generation sequencing for rapid detection, species differentiation, drug resistance profiling, and prognostic evaluation in Mycobacterium tuberculosis: a multi-center retrospective cohort study
Source: Front Cell Infect Microbiol. 2026 Aug 18;16:1818202. doi: 10.3389/fcimb.2026.1818202 (PMC13529550; doi:10.3389/fcimb.2026.1818202)
Supplement: Supplementary file 1 [file Table1.docx]

**Supplementary information for**

**Clinical Utility of Targeted Next-Generation Sequencing for Rapid Detection, Species Differentiation, Drug Resistance Profiling, and Prognostic Evaluation in Mycobacterium tuberculosis: A Multi-Center Retrospective Cohort Study**

Li Shi^1#^, Mingde Ni^2#^, Fan Yang^3#^, Wei Shi^4^, Xiuwu Liu^2^, Chen Ni^5^, Fang He^1^, Yunhui Huang^1^, XiaoyunYang^1^, Yi Pei^1*^

^1^Department of Tuberculosis, The Affiliated Changsha Central Hospital, Hengyang Medical School, University of South China, Changsha, Hunan, PR China

^2^Linyi People's Hospital, Linyi, Shandong, PR China

^3^Dinfectome Inc., Nanjing, Jiangsu, PR China

^4^School of Information Engineering, Changsha Medical University, Changsha, Hunan, PR China.

^5^The Second Clinical Medical College of Guangxi Medical University, Nanning, Guangxi, PR China

^#^Li Shi, Mingde Ni and Fan Yang have contributed equally to this work.

^*^Corresponding author: Yi Pei

E-mail address: [1113284116@qq.com](mailto:1113284116@qq.com) (Y· Pei)

**Supplementary Table S1. Statistical analysis of differences in included and excluded populations**

**Supplementary Table S2. Correspondence between tNGS sample types and clinical sample types**

**Supplementary Table S3. Statistical analysis of drug-corresponding samples in drug susceptibility testing**

**Supplementary Table S4. Pharmacological class-level coverage of the in-house mycobacterial resistance module**

**Supplementary Table S5. Detection and statistics of non-tuberculous mycobacteria (NTM) species**

**Supplementary Table S6. Drug adjustment and outcomes of patients**

**Supplementary Table S1. Statistical analysis of differences in included and excluded populations**

|  | **Variable** | **Inclusion group (N=130)** | **Exclusion group (N=20)** | **P value** |
| --- | --- | --- | --- | --- |
|  | Age (years, median, IQR) | 55.50 [35.00, 64.75] | 41.00 [34.75, 63.50] | 0.658183 |
| Gender (n, %) | | | | |
| Male | | 80 (61.5%) | 11 (55.0%) | 0.755487 |
| Female | | 50 (38.5%) | 9 (45.0%) | 0.755487 |
| TB history (n, %) | | 48 (36.9%) | 4 (20.0%) | 0.219406 |
|  | Clinical symptoms (n, %) | | | |
|  | Cough | 102 (78.5%) | 12 (60.0%) | 0.091474 |
|  | Productive cough | 90 (69.2%) | 9 (45.0%) | 0.060646 |
|  | Fever | 30 (23.1%) | 4 (20.0%) | 1 |
|  | Hemoptysis | 18 (13.8%) | 2 (10.0%) | 1 |
|  | Underlying diseases | 61 (46.9%) | 8 (40.0%) | 0.735853 |
|  | Imaging features (n, %) | | | |
|  | Cavity | 29 (22.3%) | 3 (15.0%) | 0.568795 |
|  | Calcification | 11 (8.5%) | 1 (5.0%) | 1 |
|  | Pleural thickening adhesion | 4 (3.1%) | 0 (0.0%) | 1 |
|  | Fibrosis | 1 (0.8%) | 0 (0.0%) | 1 |
|  | Nodular and linear opacities | 64 (49.2%) | 9 (45.0%) | 0.910721 |
|  | Ground-glass opacity | 3 (2.3%) | 1 (5.0%) | 0.439352 |
|  | Pleural effusion | 4 (3.1%) | 1 (5.0%) | 0.516153 |
|  | Destroyed lung | 2 (1.5%) | 0 (0.0%) | 1 |
|  | Bronchiectasis | 5 (3.8%) | 0 (0.0%) | 1 |
|  | Laboratory index (median, IQR) | | | |
|  | WBC (×10^9^/L) | 6.28 [4.55, 7.94] | 5.17 [4.31, 6.57] | 0.151102 |
|  | HGB (g/L) | 128.00 [115.00, 140.00] | 121.50 [116.50, 136.25] | 0.887055 |
|  | PLT (×10^9^/L) | 218.00 [183.00, 281.00] | 218.00 [179.00, 265.25] | 0.555153 |
|  | CRP (mg/L) | 9.00 [5.94;22.70] | 8.10 [3.31, 17.10] | 0.960798 |
|  | PCT (ng/mL) | 0.04 [0.03, 0.07] | 0.05 [0.04, 0.09] | 0.516151 |
|  | ESR (mm/h) | 22.00 [12.00, 44.75] | 28.50 [8.75, 49.00] | 0.873682 |
|  | LYMPH (×10^9^/L) | 1.25 [0.98, 1.64] | 1.17 [0.79, 1.70] | 0.992447 |
|  | NEUT (×10^9^/L) | 4.26 [2.98, 5.76] | 3.29 [2.78, 4.16] | 0.077765 |
|  | LYMPH% (100%) | 22.50 [15.40, 28.10] | 26.35 [17.27, 31.88] | 0.20352 |
|  | NEUT% (100%) | 67.20 [62.00, 76.10] | 63.10 [59.42, 70.22] | 0.078291 |

**Supplementary Table S2. Correspondence between tNGS sample types and clinical sample types**

|  | **Sample classification of tNGS** | **Number^a^** | **Sample Type of Culture** | **Number** | **Sample Type of Xpert** | **Number** |
| --- | --- | --- | --- | --- | --- | --- |
| 1 | BALF | 80 | BALF | 55 | BALF | 71 |
|  |  |  | Sputum | 49 | Sputum | 21 |
|  |  |  | Purulent fluid | 1 |  |  |
|  |  |  | Urine | 1 |  |  |
|  |  |  | Stool | 1 |  |  |
| 2 | Sputum | 47 | BALF | 1 | BALF | 71 |
|  |  |  | Sputum | 46 | Sputum | 21 |
|  |  |  | Stool | 1 |  |  |
| 3 | Cerebrospinal fluid | 1 | Cerebrospinal fluid | 1 | Stool | 1 |
| 4 | Pericardial effusion | 1 | NA^b^ | NA | Pericardial effusion | 1 |
| 5 | Pleural effusion | 1 | Sputum | 1 | Sputum | 1 |

^a^ tNGS testing was performed on one sample for each patient, with simultaneous laboratory testing, and multiple sample types were involved for each patient.

^b^Clinical culture failed or not performed.

**Supplementary Table S3. Statistical analysis of drug-corresponding samples in drug susceptibility testing**

| **Drugs** | **Total Number^a^(N=130)** | **Sputum (n=97)** | **BALF (n=56)** | **Purulent fluid (n=1)** | **Cerebrospinal fluid (n=1)** |
| --- | --- | --- | --- | --- | --- |
| Rifampicin | 52(40.0%) | 33(34.0%) | 22(39.3%) | 1(100.0%) | 1(100.0%) |
| Isoniazid | 47(36.2%) | 29(29.9%) | 20(35.7%) | 1(100.0%) | 1(100.0%) |
| Ethambutol | 51(39.2%) | 32(33.0%) | 22(39.3%) | 1(100.0%) | 1(100.0%) |
| Streptomycin | 44(33.8%) | 26(26.8%) | 20(35.7%) | 1(100.0%) | 1(100.0%) |
| Kanamycin | 39(30.0%) | 23(23.7%) | 18(32.1%) | 1(100.0%) | 1(100.0%) |
| Amikacin | 44(33.8%) | 27(27.8%) | 20(35.7%) | 1(100.0%) | 1(100.0%) |
| Clarithromycin | 39(30.0%) | 23(23.7%) | 18(32.1%) | 1(100.0%) | 1(100.0%) |
| Moxifloxacin | 43(33.1%) | 26(26.8%) | 19(33.9%) | 1(100.0%) | 1(100.0%) |
| Levofloxacin | 39(30.0%) | 23(23.7%) | 18(32.1%) | 1(100.0%) | 1(100.0%) |
| Prothionamide | 21(16.2%) | 13(13.4%) | 8(14.3%) | 0(0.0%) | 0(0.0%) |
| P-aminosalicylic acid | 22(16.9%) | 14(14.4%) | 8(14.3%) | 0(0.0%) | 0(0.0%) |
| Rifabutin | 12(9.2%) | 8(8.2%) | 3(5.4%) | 1(100.0%) | 0(0.0%) |
| Pasiniazid | 2(1.5%) | 2(2.1%) | 1(1.8%) | 0(0.0%) | 0(0.0%) |

^a^One final result per patient was presented for each drug, and some patients involved multiple sample types for culture during testing.

**Supplementary Table S4. Pharmacological class-level coverage of the in-house mycobacterial resistance module**

| **Antimicrobial class** | **No. of agents** | **Resistance-associated genes/loci** | **Applicable organisms** |
| --- | --- | --- | --- |
| Rifamycins | 1 | *rpoB* | MTBC and NTM |
| Isoniazid-related agents | 1 | *katG, inhA, fabG1* | MTBC |
| Thioamides | 2 | *inhA, fabG1* | MTBC |
| Pyrazinamide-related agents | 1 | *pncA* | MTBC |
| Ethylenediamine derivatives | 1 | *embB* | MTBC |
| Diarylquinolines | 1 | *Rv0678* | MTBC |
| Riminophenazines | 1 | *Rv0678* | MTBC |
| Folate pathway inhibitors | 1 | *folC, thyA* | MTBC |
| D-alanine pathway inhibitors | 1 | *alr* | MTBC |
| Oxazolidinones | 1 | *rplC, rrl* | MTBC |
| Aminoglycosides | 5 | *rpsL, rrs, eis* | MTBC and NTM |
| Cyclic peptide antimicrobials | 1 | *rrs* | MTBC |
| Fluoroquinolones | 3 | *gyrA, gyrB* | MTBC and NTM |
| Nitroimidazoles | 1 | *ddn* | MTBC |
| Macrolides | 2 | *erm(41), rrl* | NTM |

MTBC, Mycobacterium tuberculosis complex; NTM, nontuberculous mycobacteria. The principal evidence sources were the second edition of the WHO Catalogue of Mutations in Mycobacterium tuberculosis Complex and Their Association with Drug Resistance (WHO, 2023), ReSeqTB accessed in 2024, and relevant peer-reviewed literature available through 2024. The table presents category-level coverage rather than the complete drug-gene-variant mapping. Variants were curated according to phenotypic, functional, clinical, and consensus evidence. Variants with insufficient or conflicting evidence were classified as variants of uncertain significance and did not trigger a resistant call. The complete mutation inventory is proprietary and is not reproduced.

**Supplementary Table S5. Detection and statistics of non-tuberculous mycobacteria (NTM) species**

| **Species** | **Subspecies** | **Count** | **Sample** | **Sample Type of tNGS** | **Sample Type of Culture** | **Results of culture** |
| --- | --- | --- | --- | --- | --- | --- |
| *Mycobacterium szulgai* |  | 1 | S001 | Sputum | Sputum | *Mycobacterium tuberculosis* |
| *Mycobacterium colombiense* |  | 3 | S002 | BALF | BALF | *Mycobacterium avium* complex |
|  |  |  | S003 | Sputum | Sputum | Negative |
|  |  |  | S004 | Sputum | Sputum | *Mycobacterium avium* complex |
| *Mycobacterium abscessus* | *M. abscessus* subsp. *abscessus* | 1 | S005 | BALF | BALF | *Mycobacterium chelonae* or *M. abscessus* |
| *Mycobacterium intracellulare* |  | 1 | S006 | BALF | BALF | *Mycobacterium* |
| *Mycobacterium intracellulare* | *M. intracellulare* subsp. *intracellulare* | 14 | S007 | BALF | Sputum | Negative |
|  |  |  | S008 | BALF | BALF | *M. intracellulare* |
|  |  |  | S009 | BALF | BALF | *Mycobacterium* |
|  |  |  | S010 | BALF | BALF | NTM |
|  |  |  | S011 | BALF | BALF | NTM |
|  |  |  | S012 | Sputum | Sputum | Negative |
|  |  |  | S013 | Sputum | Sputum | Negative |
|  |  |  | S014 | Sputum | Sputum | Negative |
|  |  |  | S015 | Sputum | Sputum | Negative |
|  |  |  | S016 | Sputum | Sputum | Negative |
|  |  |  | S017 | Sputum | Sputum | Negative |
|  |  |  | S018 | Sputum | Sputum | *M. intracellulare* |
|  |  |  | S019 | Sputum | Sputum | *M. intracellulare* |
|  |  |  | S020 | Sputum | Sputum | *Mycobacterium* |

**Supplementary Table S6. Drug adjustment and outcomes of patients**

|  | | **Total (N=126)** | | **tNGS-** **dependent** | | **TB (N=87)** | | | | | **NTM (N=18)** | | | | **Other (N=17)** | | | | **Mix (N=4)** | | |
| --- | --- | --- | --- | --- | --- | --- | --- | --- | --- | --- | --- | --- | --- | --- | --- | --- | --- | --- | --- | --- | --- |
|  |  |  |  |  |  | **Total** | | | **tNGS-dependent** | | **Total** | | **tNGS-dependent** | | **Total** | | **tNGS-dependent** | | **Total** | | **tNGS-dependent** |
| **Therapy adjustment (n, %)** | | | | | | | | | | | | | | | | | | | | | |
| Initiation | 36 (28.57%) | | 24 (66.67%) | | 30 (34.48%) | | | 19 (66.33%) | | 4 (22.22%) | | 4 (100.00%) | | 2 (11.76%) | | 1 (50.00%) | | - | | - | |
| Escalation | 7 (7.14%) | | 4 (57.14%) | | 4 (4.60%) | | | 2 (50.00%) | | 3 (16.67%) | | 2 (66.67%) | | - | | - | | - | | - | |
| De-escalation | 1 (0.79%) | | 1 (100.00%) | | 1 (1.15%) | | | 1 (100.00%) | | - | | - | | - | | - | | - | | - | |
| Maintenance | 44 (34.92%) | | 0 (0.00%) | | 24 (27.59%) | | | 0 (0.00%) | | 6 (33.33%) | | 0 (0.00%) | | 12 (70.59%) | | 0 (0.00%) | | 2 (50.00%) | | 0 (0.00%) | |
| Replacement | 34 (26.98%) | | 19 (55.88%) | | 25 (28.74%) | | | 13 (52.00%) | | 5 (27.78%) | | 3 (60.00%) | | 3 (17.65%) | | 2 (66.67%) | | 1 (25.00%) | | 1 (100.00%) | |
| Discontinuation | 4 (3.17%) | | 4 (100.00%) | | 3 (3.45%) | | | 3 (100.00%) | | - | | - | | - | | - | | 1 (25.00%) | | 1 (100.00%) | |
| **Outcome (n, %)** | | | | | | | | | | | | | | | | | | | | | |
| Cured | 1 (0.79%) | | 0 (0.00%) | | - | | - | | | - | | - | | 1 (5.88%) | | 0 (0.00%) | | - | | - | |
| Improved | 99 (78.57%) | | 43 (43.43%) | | 71 (81.61%) | | 31 (43.66%) | | | 11 (61.11%) | | 7 (63.64%) | | 13 (76.47%) | | 3 (23.08%) | | 4 (100.00%) | | 2 (50.00%) | |
| No improvement | 13 (10.32%) | | 5 (38.46%) | | 6 (46.15%) | | 3 (50.00%) | | | 5 (38.46%) | | 2 (40.00%) | | 2 (15.38%) | | 0 (0.00%) | | - | | - | |
| Deteriorated | 3 (2.38%) | | 1 (33.33%) | | 2 (2.30%) | | 1 (50.00%) | | | 1 (5.56%) | | 0 (0.00%) | | - | | - | | - | | - | |
| Unknown | 10 (7.94%) | | 3 (30.00%) | | 8 (80.00%) | | 3 (37.50%) | | | 1 (10.00%) | | 0 (0.00%) | | 1 (10.00%) | | 0 (0.00%) | | - | | - | |
